# Revealing the Diverse Allergenic Protein Repertoire of Six Widely Consumed Crab Species: A Species‐Specific Allergen in King Crab

**DOI:** 10.1111/all.16674

**Published:** 2025-07-30

**Authors:** Shanshan Li, Jingyuan Bian, Qing Xiong, Brian Shing‐Hei Wong, Stephen Kwok‐Wing Tsui, Kin‐Ming Kwan, Nicki Yat‐Hin Leung, Ting‐Fan Leung, Patrick S. C. Leung, Ka‐Hou Chu, Xiaojun Xiao, Christine Yee‐Yan Wai

**Affiliations:** ^1^ School of Life Sciences The Chinese University of Hong Kong Hong Kong SAR China; ^2^ Institute of Allergy and Immunology of Shenzhen University School of Medicine State Key Laboratory of Respiratory Disease Allergy Shenzhen University Division Shenzhen China; ^3^ Department of Health Technology and Informatics The Hong Kong Polytechnic University Hong Kong SAR China; ^4^ School of Biomedical Sciences The Chinese University of Hong Kong Hong Kong SAR China; ^5^ Hong Kong Bioinformatics Centre The Chinese University of Hong Kong Hong Kong SAR China; ^6^ Department of Paediatrics Prince of Wales Hospital, the Chinese University of Hong Kong Hong Kong SAR China; ^7^ Hong Kong Hub of Paediatric Excellence The Chinese University of Hong Kong Hong Kong SAR China; ^8^ Division of Rheumatology, Allergy and Clinical Immunology University of California Davis California USA; ^9^ Southern Marine Science and Engineering Guangdong Laboratory (Guangzhou) Guangzhou China

**Keywords:** allergen comparison, crab allergen, king crab, novel allergen, shellfish allergy

## Abstract

**Background:**

Shellfish allergy poses a significant health risk affecting up to 2% of the global population. Comprehensive allergen profiling across species is crucial for improving diagnostics and therapies, given the challenges posed by cross‐reactivity. This study aims to identify and compare the allergen profiles of six widely consumed edible crab species.

**Methods:**

Muscle proteins were extracted from five brachyurans (true crabs) including *Charybdis feriata*, *Portunus pelagicus* , *Scylla paramamosain*, *Chionoecetes opilio* , and *Eriocheir sinensis* , as well as the king crab *Paralithodes camtschaticus* , and were analyzed for IgE reactivity with serum samples from 29 crab‐allergic individuals and three nonallergic controls. IgE‐binding proteins were identified by immunoblotting followed by mass spectrometry. Recombinant king crab allergen was purified and tested on ELISA against samples from 50 crab‐allergic individuals, with its specific IgE reactivity evaluated by inhibition ELISA and immunoblot. Comparison of the gene expression of the identified allergens along with reported epitopes was revealed through comparative transcriptomics and multiple sequence alignments.

**Results:**

IgE reactivity was detected only in serum samples from crab‐allergic individuals. Immunoblotting distinguished eight putative crab allergens and three registered crab allergens. The protein and allergen profiles of the king crab were distinct from the brachyuran crab species based on dendrogram analysis; malate dehydrogenase (MDH) was distinctly reactive only in king crab with 41.4% sensitization on immunoblot, while recombinant MDH displayed a 14% sensitization rate, leading to its registration as Para c 11. MDH homologs from true crabs showed minimal inhibition to Para c 11 (< 10%). Based on transcriptomic analysis, the identified crab allergens showed similar expression across species, while the sequence and epitope similarity exceeded 68%.

**Conclusion:**

The study provides molecular insights into crab allergen diversity and highlights the potential for species‐specific crab allergies with Para c 11 as a potential king crab‐specific allergen, paving the way for personalized and advanced component‐resolved diagnostics.

## Graphical abstract

This study comprehended the allergen profiles of six edible crab species using proteomic and transcriptomic analyses and identified 11 putative allergens. King crab has a distinct protein and allergen profile, with the discovery of malate dehydrogenase as a novel king crab‐specific allergen registered as Para c 11. The distinct profiles and allergen repertoire between king crab and true crabs highlight the clinical need to refine the diagnostic strategies of crab allergy.

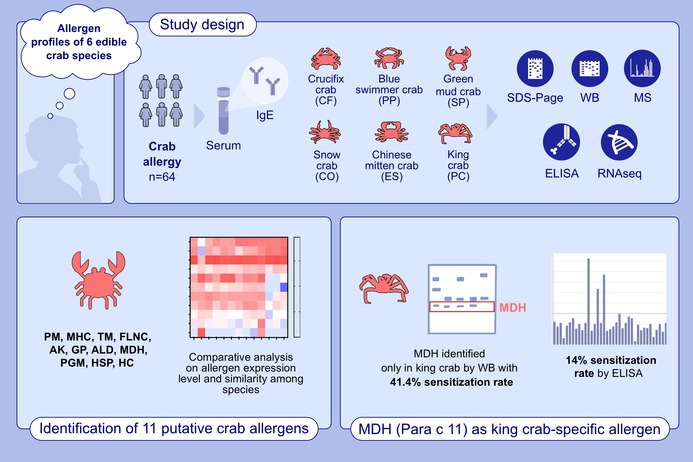

**Abbreviations**
AK: arginine kinase
ALD: aldolase
CF: *Charybdis feriata*
CO: *Chionoecetes opilio*
ELISA: enzyme‐linked immunosorbent assay
ES: *Eriocheir sinensis*
FLNC: filamin C
GP: glycogen phosphorylase
HC: hemocyanin
HSP: heat shock protein
MDH: malate dehydrogenase
MHC: myosin heavy chain
MS: mass spectrometry
PC: *Paralithodes camtschaticus*
PGM: phosphoglucomutase
PM: paramyosin
PP: *Portunus pelagicus*
RNAseq: RNA sequencing
SDS‐PAGE: sodium dodecyl sulfate polyacrylamide gel electrophoresis
SP: *Scylla paramamosain*
TM: tropomyosin
WB: western blot

## 1 Introduction

Shellfish allergy is one of the most prevalent food allergies with a growing health issue globally [1, 2, 3, 4]. It is the most common (2.9%) food allergy among adults and the third most common (1.3%) among children in the United States [3, 5], and is one of the leading causes of food allergy in most Asian countries [6, 7, 8]. Shellfish allergies are notable for their potential to cause severe allergic reactions and lifelong persistence without outgrowing with age [4, 9]. Among shellfish allergies, crabs are the second most frequent causative food following shrimp [10, 11].

Studies on shellfish allergies estimated that approximately 75% of affected individuals exhibit sensitivities to all species of Crustacea, while a subset of individuals displays selective reactivity toward a limited number of species [12, 13, 14]. Nevertheless, the diversity of edible seafood poses significant challenges in accurately identifying the precise species and allergenic components that elicit allergic reactions. Consequently, a precautionary approach of complete shellfish avoidance is commonly recommended to mitigate potential risks. Identifying the precise allergens capable of eliciting food hypersensitivity reactions is of utmost importance for effectively managing allergies. Such knowledge facilitates the development of more sophisticated diagnostic techniques, more focused therapeutic interventions, and possible preventative measures, including immunotherapy or allergen‐directed remedies.

To date, several crab‐derived allergens have been registered in the World Health Organization and International Union of Immunological Societies (WHO/IUIS) Allergen Nomenclature Database, including tropomyosin (TM) [15, 16, 17], arginine kinase (AK) [18, 19], myosin light chain (MLC) [20], sarcoplasmic calcium‐binding protein (SCP) [21], triosephosphate isomerase (TIM) [22], and filamin C (FLNC) [23]. Despite the increasing global consumption of crabs [24], comprehensive comparative analyses of allergenic proteins across different crab species remain limited.

**FIGURE 1:**
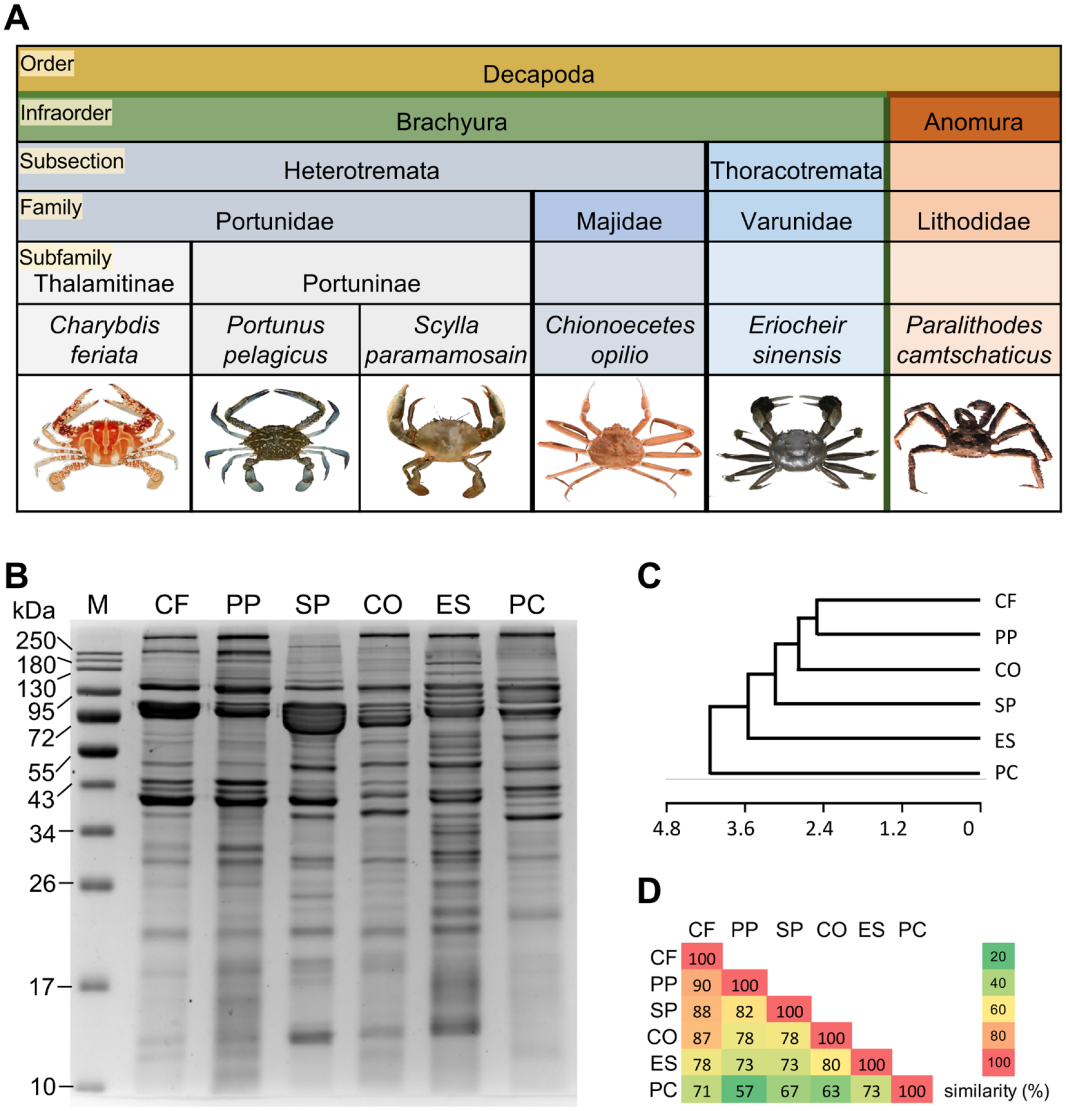
Crab species under study and their muscle protein profile. (A) Taxonomy of the six crab species based on the classification system of the NCBI database. (B) SDS‐PAGE of crab muscle protein. 4%–10% SDS‐PAGE of whole muscle extracts stained with Coomassie brilliant blue. CF, *Charybdis feriata*; CO, *Chionoecetes opilio* ; ES, *Eriocheir sinensis* ; M, molecular weight markers; PC, *Paralithodes camtschaticus* ; PP, *Portunus pelagicus* ; SP, *Scylla paramamosain*. (C) Dendrogram of six species based on SDS‐PAGE. (D) Similarity index of protein bands observed in SDS‐PAGE among the crab species.

In this study, six crab species spanning two infraorders within Decapoda, Brachyura (true crab) and its sister taxon Anomura [25], were selected to represent the key species consumed widely (Figure 1A). Five brachyuran species were analyzed: (1) *Charybdis feriata* (family Portunidae), a species of swimming crab commonly known as crucifix crab, which is native to the Indo‐Pacific region and extends from East Africa to Japan, Indonesia, and Australia; (2) *Portunus pelagicus* (family Portunidae), the blue swimmer crab, which supports large fisheries throughout the Indo‐Pacific and is a delicacy across Asia; (3) *Scylla paramamosain* (family Portunidae), a mud crab found along the coast of the South China Sea down to the Java Sea, cultured in China and southern Vietnam [26], and commonly consumed in the region; (4) *Chionoecetes opilio* (family Majidae), snow crab inhabiting the Northwest Atlantic and North Pacific shelves, where it represents an economically crucial commercial fishery; and (5) *Eriocheir sinensis* (family Varunidae), the Chinese mitten crab, inhabiting rivers and coasts of China, with cultural significance as a domestically popular food in the country [27]. In addition, a representative from Anomura, the red king crab *Paralithodes camtschaticus* (family Lithodidae, abbreviated as *Para. camtschaticus* to avoid confusion with species from the genus *Portunus*) was included in the study. The red king crab, indigenous to the North Pacific and adjacent seas, has been introduced to the Barents Sea; it stands as one of the world's premier crabs in the global market [28].

This study aims to conduct a systematic molecular characterization and inter‐species comparison of crab allergens. The detailed allergen repertoire of the above six frequently consumed crab varieties was investigated via comparative IgE‐binding analysis. Subsequently, the expression level, sequence, and epitope similarity of the identified homologous proteins of the crab putative allergens were analyzed through bioinformatics. Overall, the analysis provides comprehensive allergen profiles across crab species and sheds new light on the potential management for species‐specific crab allergies.

## 2 Materials and Methods

### 2.1 Preparation of Protein Extracts

Live whole *C. feriata*, *P. pelagicus* , *S. paramamosain*, *C. opilio* , *E. sinensis* , and *Para. camtschaticus* specimens were purchased at a local market (Taipo, Hong Kong), and proteins were extracted from muscle tissue following a standardized protocol. Muscle tissue from the claws of each crab was minced and mixed in PBS (pH 7.4) at a ratio of 4 mL per gram at 4°C overnight. The mixture was then centrifuged at 5000 *g* for 20 min at 4°C. Following centrifugation, the supernatant was filtered (0.2 μm pore size), collected, and stored at −20°C until analysis. The protein concentration of each extract was determined with a BCA protein assay kit (Thermo Fisher Scientific, IL, USA) using bovine serum albumin as a standard.

### 2.2 Protein Profiling by SDS‐PAGE

Ten micrograms of each protein extract were resolved under reducing conditions on a 12% separating gel paired with a 4% stacking gel together with pre‐stained protein ladder (New England Biolabs, MA, USA) as molecular weight standard run alongside. Gels were stained with SimplyBlue SafeStain (Invitrogen, CA, USA) and then scored based on a polypeptide bands' presence (+) or absence (−). Dendrogram construction was carried out utilizing the SRplot platform [29]. The similarity index (SI) between species was computed via the formula:$\mathrm{SI}=\left(2Z/\left(X+Y\right)\right)\times 100$,where Z is the number of matching bands and *X*+*Y* is the total bands compared.

### 2.3 Crab‐Allergic Subjects

Serum samples for this study were obtained from 64 crab‐allergic subjects (39 males and 25 females) with a mean age of 18.3 years (range: 1–55 years) (Table S1). All allergic subjects were identified from Prince of Wales Hospital, Hong Kong, based on their clinical history of immediate allergy to crabs (i.e., allergic reactions within 2 h of crab ingestion). Sera were also collected from three nonallergic subjects as negative controls. Demographic data of the recruited subjects as well as their clinical history were collected with a standardized questionnaire. Skin prick tests were performed using shellfish mix (ALK‐Abelló) according to the manufacturer's instructions, with histamine and normal saline as controls. Specific IgE to shrimp (f24) and crab (f23) were measured on a Phadia 200 system. Participants and/or their parents gave written informed consent. Ethics approval for this study was obtained from the Joint Chinese University of Hong Kong—New Territories East Cluster Clinical Research Ethics Committee (2018.484), Hong Kong.

### 2.4 Immunoblotting

Equal amounts of extracted proteins or 2 μg/lane of purified recombinant proteins were resolved by SDS‐PAGE and subsequently transferred from the gel onto a PVDF membrane (Bio‐Rad, CA, USA) using the Trans‐Blot Turbo system (Bio‐Rad). The membrane was then blocked for 1 h at room temperature (RT) with 5% skim milk/TBS (blocking buffer, Bio‐Rad) and thereafter probed with patient sera diluted at 1:10 in blocking buffer and incubated overnight at 4°C with shaking using the Surf‐Blot system (Idea Scientific, MN, USA). The membrane was then washed four times for 10 min each with TBS‐T (TBS buffer containing 0.05% Tween‐20), followed by incubation with HRP‐conjugated antihuman IgE (Southern Biotech, Birmingham, AL, USA) diluted at 1:2000 in blocking buffer. After six washes with TBS‐T, the membrane was incubated with SuperSignal West Pico PLUS Chemiluminescent Substrate (Thermo Fisher Scientific, MA, USA) for 2 min. IgE‐reactive bands to each serum sample were visualized using the ChemiDoc MP Imaging System (Bio‐Rad) [30]. Dendrogram construction was performed using the SRplot platform, with similarity matrices calculated based on the Pearson coefficient to compare sets of variables [31].

### 2.5 Mass Spectrometry (MS)

Regions corresponding to the IgE‐binding bands were excised from SDS‐PAGE gels and subjected to trypsin digestion to enzymatically cleave the proteins into smaller peptide fragments as per prior protocols [32]. The resultant peptides were then analyzed utilizing the UltrafleXtreme MALDI ToF/ToF System (Bruker Daltonics GmbH, Bremen, Germany). Samples (0.5 μL) were spotted onto an MTP AnchorChip plate (Bruker Daltonics GmbH), followed by matrix solution (1 mg/mL α‐cyano‐4‐hydroxycinnamic acid in 95% acetonitrile, 0.1% trifluoroacetic acid). Mass spectra were acquired in the reflectron positive‐ion mode from 4000 laser shots with an acceleration of 20 kV. The mass spectra were internally calibrated using trypsin autolytic products, yielding mass errors below 10 ppm. Precursor ions with an S/N ratio ≥ 10 were selected for tandem mass spectrometry (accumulated from 6000 laser shots). The spectra data were searched by ProteinScape software 3.0 using MASCOT (Matrix Sciences, London, UK) as a search engine against the NCBI and Uniprot database (product ion mass tolerance set at 0.5 Da).

### 2.6 Expression and Purification of Recombinant Proteins

Malate dehydrogenase (MDH) of *Para. camtschaticus* (Accession No. PP541528) was cloned and expressed in pET‐22b (+) plasmid with an N‐terminal His6‐tag, whereas MDH of *S. paramamosain* and *E. sinensis* was cloned and expressed in pET30a (+) plasmid with both N‐ and C‐terminal His6‐tags. MDH homologs from *S. paramamosain* and *E. sinensis* were selected due to their highest and lowest homologies with MDH of *Para. camtschaticus* (i.e., 89.1% and 96.8%, respectively) to access cross‐reactivity. The recombinant plasmids were transformed into BL21(DE3) *E. coli* , and protein expression was induced by isopropyl ß‐D‐1‐thiogalactopyranoside (IPTG). Cells were lysed, and the soluble proteins were purified using Ni‐NTA affinity chromatography under denaturing conditions. The purified proteins were then refolded by stepwise dialysis. Protein purity of the recombinant MDHs (rMDHs) was determined using SDS‐PAGE, while the concentration was determined using BCA Protein Assay, with BSA as a reference. The molecular weight and purity of each recombinant protein were determined with SDS‐PAGE.

### 2.7 Enzyme‐Linked Immunosorbent Assay (ELISA)

ELISA for detecting serum‐specific IgE was conducted as described previously [30]. Specifically, rMDH (5 μg/mL) was coated onto ELISA plates and incubated overnight at 4°C. The plates were blocked with 5% fetal bovine serum (Gibco, Thermo Fisher Scientific) diluted in PBS (blocking buffer) at RT for 2 h, and then incubated with 100 μL of diluted sera collected from 50 crab‐allergic patients at a ratio of 1:10 (v/v) in blocking buffer overnight at 4°C. Plates were washed and incubated with 100 μL biotin‐antihuman IgE (Vector labs, CA, USA, 1:1000 dilution in blocking buffer) followed by 100 μL Horseradish Avidin D (Vector labs, 1:1000 dilution in blocking buffer). Finally, 100 μL of 3,3′,5,5′‐tetramethylbenzidine substrate (BD Biosciences, NJ, USA) was added, and color development was stopped by adding 50 μL of 0.1 M sulfuric acid after 30 min. Color intensity was measured using a microplate reader (BioTek, CA, USA) at 450 nm.

### 2.8 Inhibition Assays

Inhibition ELISA assay was performed as described above, with modifications to assess cross‐reactivity of rMDH from *Para. camtschaticus* (PC‐MDH). A pooled serum sample (1:10 v/v) from crab‐allergic individuals (*n* = 9, selected based on confirmed IgE reactivity PC‐MDH) was preincubated for 2 h at 30°C with serial dilutions of rMDH from *S*. *paramamosain* (SP‐MDH), *E. sinensis* (ES‐MDH), or *Para. camtschaticus* (0.0001 to 100 μg/mL), followed by overnight incubation at 4°C on 96‐well plates coated with 5 μg/mL of PC‐MDH. The percentage of inhibition was calculated using the formula:$\left[\left(\mathrm{OD}\;\mathrm{no}\;\text{inhibition}/\mathrm{OD}\;\text{inhibition}\right)/\mathrm{OD}\;\mathrm{no}\;\text{inhibition}\right]\times 100\%$.

To further study the cross‐reactivity between PC‐MDH and MDH homologs from other crab species, an IgE immunoblot inhibition experiment was also performed. SDS‐PAGE‐resolved PC‐MDH (2 μg/lane) was transferred to a PVDF membrane (Bio‐Rad). After blocking, membrane strips were incubated overnight at 4°C with pooled sera (1:10 v/v) from crab‐allergic patients (*n* = 9) preincubated for 2 h at 30°C with rMDHs (SP‐MDH or ES‐MDH) at serial concentrations between 0.0001 and 100 μg/mL. Detection and visualization were performed as described in the general immunoblotting protocol. Band intensity was quantified using ImageJ software, and the percentage of inhibition was calculated as follows: $\begin{array}{c} {}[(\text{Intensity without inhibitor}\;-\;\text{Intensity with inhibitor})/ \end{array}$ $\begin{array}{c} \text{Intensity without inhibitor}]\times 100\% \end{array}$.

### 2.9 Basophil Activation Test

Basophil activation test (BAT) was performed to assess the functional IgE reactivity of rMDH from *Para. camtschaticus* according to our published protocol [33]. Briefly, in vitro basophil activation was quantified using the Flow CAST kit (Flow CAST kit; BÜHLMANN Laboratories) as per the manufacturer's instructions. EDTA‐anticoagulated venous blood was collected from two crab‐allergic subjects who showed a positive IgE response to PC‐MDH. Whole blood was then incubated with serial concentrations of extracts or rMDH from *Para. camtschaticus, S. paramamosain*, and *E. sinensis* (100, 1000, 50,000, and 10,000 ng/mL). Stained cells were acquired on a BD LSRFORTESSA flow cytometer with basophils gated as CCR3^pos^/SSC^low^. Upregulation of the basophil marker CD63 was calculated based on the percentage of CD63^+^ cells compared with the total number of identified basophils. In each assay, a minimum of 500 events (i.e., CCR3^pos^ basophils) were recorded.

### 2.10 Transcriptome Profiling and Expression Level Analysis

A multifaceted approach was employed to acquire reference coding and accurate sequences of the 11 identified genes across the six crab species. The annotated NCBI GenBank assemblies, GCA_035594125.1 for *S. paramamosain* and GCA_024679095.1 for *E. sinensis* [34, 35], served as the foundational reference genomes. De novo transcriptome assemblies for *C. feriata* and *C. opilio* were constructed using RNA‐sequencing data (*C. feriata*: RNA sequencing of muscle tissue generated with Illumina Hiseq‐PE150, raw reads: 71,249,362; *C. opilio* : RNA sequencing of mixed tissue from hepatopancreas, gonad, gill, and muscle generated with Illumina Hiseq‐PE150, raw reads: 42,205,765) by Trinity v2.15.2. In addition, RNA‐sequencing data downloaded from NCBI SRA, including SRR6214447 for *C. feriata* [36], SRR8668058 for *P. pelagicus* [36], SRR14684335 for *E. sinensis* [37], and a combination of ERR5845122, ERR5845127, and SRR26363645 for *Para. camtschaticus* [38, 39], were harnessed to generate further de novo transcriptome assemblies with Trinity. PacBio long‐read sequencing data supplemented the transcriptomic resources for *P. pelagicus* [40], while the Crustacean Annotated Transcriptome database [41] provided alternative references for *Para. camtschaticus*.

Homologous sequences among the six species were identified using BLAST 2.9.0+, and multiple sequence alignment results were generated with the Clustal Omega program conducted in Jalview v2.11.3.3 [42]. RNA‐sequencing data were collected to quantify the expression levels. Briefly, the mapping‐based mode of Salmon v0.14.1 was used for data analysis. The raw RNA‐sequencing data of *C. feriata* and *C. opilio* from this study, along with SRA data including SRR8668058 for *P. pelagicus* [37]; SRR23867489, SRR23867491, SRR23867492, and SRR23867493 for *S. paramamosain* [43]; SRR14684335, SRR14684346, SRR14684347, SRR7984118, SRR7984119, and SRR7984120 for *E. sinensis* [44, 45]; and ERR5845122, ERR5845127, and SRR10023617 for *Para. camtschaticus* [38, 41], were included in the analysis. RNA‐seq data available for *P. pelagicus* and *C. opilio* were obtained from mixed tissue sources, including hepatopancreas, gonad, gill, and muscle, while the transcriptome data for the other four species were explicitly derived from claw muscle tissue. The transcript per million (TPM) values generated by Salmon were further refined, including a log2 transformation to normalize the data and scale across columns for comparability. The processed data were then plotted with a heat map using GraphPad Prism v9.4.1.

### 2.11 Similarity Analysis of Protein and Epitope Sequences

The consensus sequences of proteins or epitopes were generated by SnapGene v7 with a threshold of at least 4 (> 50%). Similarity values between each protein and consensus sequence were calculated with the BLOMSUM62 scoring matrix, and different amino acids scored above one were considered similar.

### 2.12 Statistical Analyses

Statistical analyses were performed using GraphPad Prism v9.4.1. Comparison of TPM values among the 11 identified allergen groups in *S. paramamosain*, *E. sinensis* , and *Para. camtschaticus* (species with more than two sets of transcriptome data) and comparison of similarity values among the allergen groups were performed using one‐way ANOVA followed by Turkey's multiple comparisons test. Differences were regarded as statistically significant at *p* < 0.05.

## 3 Results

### 3.1 Characteristics of Recruited Subjects

The recruited subjects were defined as having probable crab allergy with a history of immediate allergic reactions to crab and having a class 2 or above of crab‐specific IgE level response to ImmunoCAP Allergen f23 (Thermo Fisher Scientific, IL, USA) (i.e., > 0.7 kUA/L) [46], which detects IgE binding to whole allergen extract from *C. opilio* . Shrimp‐specific IgE levels ranged from 1.1 to 90.5 kUA/L (median: 9.3 kUA/L), while crab‐specific IgE levels ranged from 1.1 to 96.8 kUA/L (median: 7.9 kUA/L). The three most common allergic symptoms reported were eczema (56.3%), angioedema (54.7%), and itchy throat (48.4%). All subjects reported allergy to crabs, with 30 (46.9%) also reporting allergy to king crab. Six (#1, 2, 5, 26, 36, and 56) tolerated king crab, while the other 28 had not tried king crab before.

### 3.2 Protein Profile of Six Crab Species

Analysis of protein extracts by SDS‐PAGE (Figure 1B) revealed an array of proteins ranging from around 10 to 250 kDa. Some inter‐species differences emerged, where most prominently, *C. feriata* and *P. pelagicus* exhibited closely aligned band distributions relative to the other species. A prominent 34–43 kDa cluster was present in all species, aligned with the known crab allergens TM and AK [15, 47, 48, 49]. Another 72–90 kDa cluster, containing a band consistent with hemocyanin (HC), showed interspecific variation in band thickness suggestive of divergent expression levels. In addition, several other bands were observed at positions that coincided with the known shellfish allergens glycogen phosphorylase (GP), MLC, SCP, and troponin C (TNC). The dendrogram (Figure 1C) showed that the branch of *Para. camtschaticus* stood out as a distinct cluster separated from the other five species, indicating its substantial divergence from the brachyuran crabs. Further, the highest SI was observed between *C. feriata* and *P. pelagicus* , reaching 90% (Figure 1D). In contrast, the SI between *Para. camtschaticus* and the other species was lower, ranging from 57% to 73%.

### 3.3 IgE Binding Pattern to Crab Allergens

**FIGURE 2:**
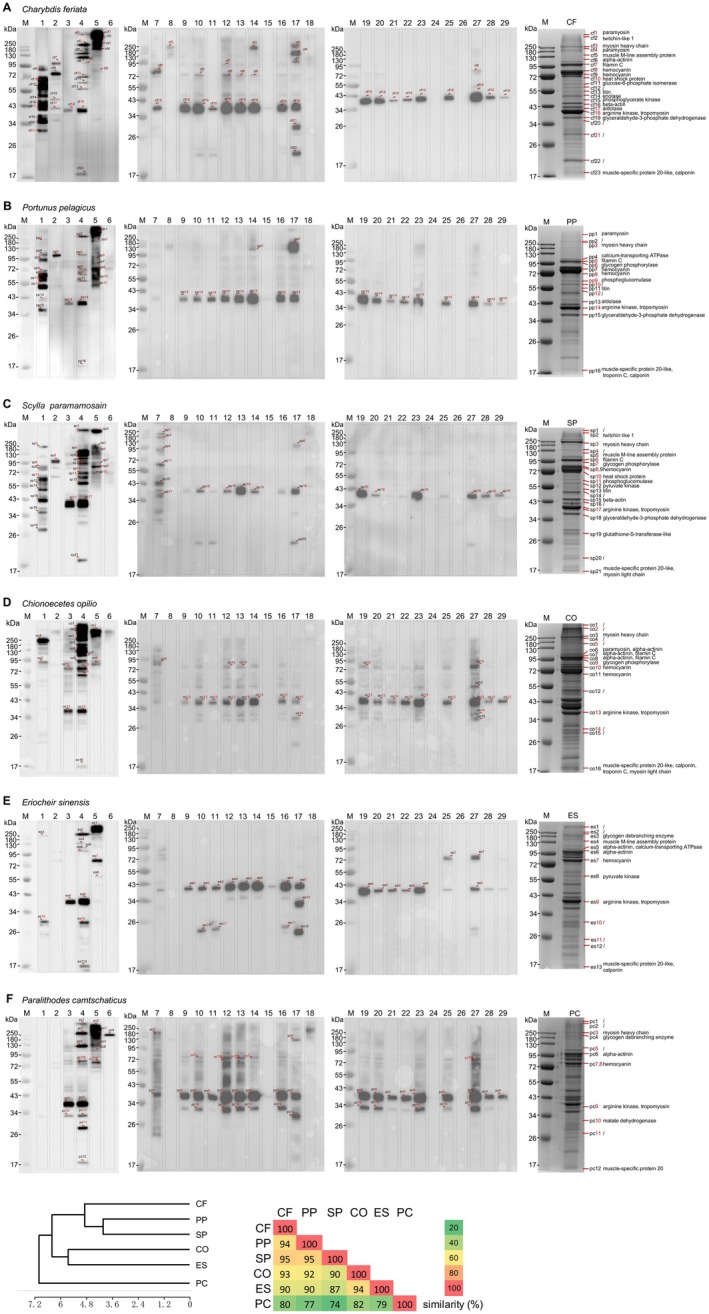
Allergen profiles of the six crab species revealed by immunoblotting. Immunoblot images for (A) *Charybdis feriata*, (B) *Portunus pelagicus* , (C) *Scylla paramamosain*, (D) *Chionoecetes opilio* , (E) *Eriocheir sinensis* , and (F) *Paralithodes camtschaticus* . SDS‐PAGE gel of protein extracts was transferred onto PVDF membrane which was probed against sera from subjects with crab allergy for IgE reactivity. Lane M, molecular weight markers in kDa (kilodaltons). The patient numbers correspond to Table S1. The labeled bands reactive to more than one serum sample were excised for protein identification by MS. Serum samples from two nonallergic subjects exhibited no IgE reactivity (data not shown). (G, H) Dendrogram and similarity index of IgE‐binding bands on immunoblot specific to different serum samples among the six crab species. CF, *Charybdis feriata*; CO, *Chionoecetes opilio* ; ES, *Eriocheir sinensis* ; PC, *Paralithodes camtschaticus*; PP, *Portunus pelagicus*; SP, *Scylla paramamosain*.

IgE binding was analyzed by immunoblotting using sera from 29 crab‐allergic individuals with specific IgE to crab > 3.5 kUA/L on ImmunoCAP allergen f23 (samples #1–29, Table S1). Differential IgE binding to crab proteins was observed among the different crab extracts, with 22 IgE‐binding bands in *C. feriata*, 16 in *P. pelagicus* , 21 in *S. paramamosain*, 16 in *C. opilio* , 13 in *E. sinensis* , and 12 in *Para. camtschaticus* (Figure 2). Most IgE‐reactive bands were higher molecular weight proteins in all extracts (> 30 kDa). Individual patients showed different IgE binding to various crab extracts. Strong and frequent IgE binding was observed in crab proteins with molecular weight between 30 and 42 kDa for all crabs. To examine the IgE‐binding pattern, positive bands of each species were compiled to construct a dendrogram (Figure 2G). It was apparent that the branch of *Para. camtschaticus* stood out as a distinct cluster separated from the other five species that was concordant with the dendrogram analysis based on SDS‐PAGE, indicating its substantial divergence from the brachyurans in the context of both protein and allergen profiles. *C. feriata*, *P. pelagicus* , and *S. paramamosain* were grouped in a separate cluster. The highest SI was observed between *P. pelagicus* and *S. paramamosain*, reaching 95% (Figure 2H). In contrast, the SI between *Para. camtschaticus* and the other species ranged from 74% to 80%, which further indicated a distinct allergen profile of *Para. camtschaticus* when compared with the true crabs.

IgE‐reactive proteins were distinguished using MS, and searches were performed against the crustacean database (UniProt) using Mascot to identify potential crustacean allergens. Only hit proteins in MS identification with a score > 30 and a matched molecular weight are shown in Table S2. MS characterized the proteins from most of the reactive bands, including paramyosin (PM), twitchin‐like 1, myosin heavy chain (MHC), glycogen debranching enzyme (GDE), muscle M‐line assembly protein, alpha‐actinin (ACTN), calcium‐transporting ATPase, FLNC, GP, HC, heat shock protein (HSP), glucose 6‐phosphate isomerase (GPI), phosphoglucomutase (PGM), pyruvate kinase (PK), titin, enolase, phosphoglycerate kinase (PGK), beta‐actin (ACTB), aldolase (ALD), AK, TM, glyceraldehyde‐3‐phosphate dehydrogenase (GPDH), MDH, muscle‐specific protein 20 like (MSP20), and MLC. Eleven IgE‐binding proteins, including PM, MHC, FLNC, GP, HC, HSP, ALD, PGM, AK, TM, and MDH (Table 1), that exhibited positive responses to two or more serum samples were identified as putative allergens for further investigations. Notably, strong IgE binding specific to AK and TM was frequently observed in all crab species, with sensitization rates ranging from 44.8% to 69.0%. In contrast, the profiles of the remaining nine proteins varied across the six species. Of particular interest, strong IgE binding to MDH with high sensitivity (41.4%) was specifically observed only in king crab (PC).

**TABLE 1:** In‐gel tryptic digest mass spectrometric identification of IgE‐binding proteins (reactive to more than two serum samples) isolated from SDS‐PAGE bands and their sensitization rate in Western blot.

*Charybdis feriata*							
Band	MW on SDS‐PAGE (kDa)	Protein name	Identified peptide	Protein accession number and Organism #	Protein MW (kDa)	Score	Sensitization rate
cf1	> 250	Paramyosin	RQAISLQEIQAHYDEIQRQ RQLQTTLDQYGVAQRR	A0A8J4YAP8 KAG0719394.1 *Chionoecetes opilio*	102.2	60.57	6.9%
cf3	223	Myosin heavy chain	RYPIYTNRT RNYATELFRS RGQLELSQVRQ	A0A8J4XWP0 KAG0710435.1 *Chionoecetes opilio*	165.8	142.7	17.2%
cf4	200	Paramyosin	RLKHDLELEIRR RQAISLQEIQAHYDEIQRQ RQLQTTLDQYGVAQRR	A0A8J4YAP8 KAG0719394.1 *Chionoecetes opilio*	102.2	105.6	6.9%
cf7	100	Filamin C	KKLPNGHLGISFTPRE RVHAGGPGLERG KVYITPSLGEARK KFNGIHIPGSPFRL	A0A5J6X3F8 QFI57017.1 *Scylla paramamosain*	91.1	155.9	13.8%
cf8	76	Hemocyanin	KGESFFWVHHQLTVR FALPPGVLEHFETATR YDNNHVEFSFNEGR	A0A0U1ZZD3 AKC96430.1 *Scylla paramamosain*	77.8	211	24.1%
cf10	70	Heat shock protein	DAVVTVPAYFNDSQR SINPDEAVAYGAAVQAAILR MVNHFAQEFQR	A0A8J4YVP5 KAG0727865.1 *Chionoecetes opilio*	70	172.3	6.9%
cf17	42	Aldolase	KGVVPLMGSEGESTTQGLDDLSQRC KYTPQQAAEATVLALSRT RKPWSLTFSYGRA KNTPSYQGMLENANVLARY	A0A8J5BSY0 KAG0703349.1 *Chionoecetes opilio*	39.6	294.4	6.9%
cf18‐1	40	Arginine kinase	KLIDDHFLFKE RIISMQMGGDLGQVYRR RLVSAVNEIEKRV RVPFSHHDRL RGEHTEAEGGIYDISNKR	Q9NH48 AAF43437.1 *Eriocheir sinensis*	43.4	404	69.0%
cf18‐2	40	Tropomyosin	RIQLLEEDLERS	A0A6C0N3G5 QHW05413.1 *Scylla paramamosain*	32.8	35.9	
cf21	28	/	/	/	/	/	6.9%

*Portunus pelagicus*							
Band	MW on SDS‐PAGE (kDa)	Protein name	Identified peptide	Protein accession number and Organism #	Protein MW (kDa)	Score	Sensitization rate
pp3	223	Myosin heavy chain	TLNATHPHFIRC RDLEEINIQHEAALSQLRK	A0A5B7DQZ0 KAG0710435.1 *Chionoecetes opilio*	218.8	68.6	6.9%
pp5	100	Filamin C	KKLPNGHLGISFTPRE KVYITPSLGEARK KFNGIHIPGSPFRL	A0A5J6X3F8 QFI57017.1 *Scylla paramamosain*	90.9	148.7	13.8%
pp6	95	Glycogen phosphorylase	KARPEYMIPVNFYGRV KFFNDGDYIQAVLDRN RILVDIEGLTWARA KLKPLVNDSGFIRT KVIYLENYRV KLPAPHEPRE	A0A0P4VYA7 JAI58945.1 *Scylla olivacea*	97.8	210.1	6.9%
pp8	76	Hemocyanin	KGESFFWVHHQLTVR FALPPGVLEHFETATR YDNNHVEFSFNEGR	A0A0U1ZZD3 AKC96430.1 *Scylla paramamosain*	77.8	141.6	10.3%
pp9	65	Phosphoglucomutase	KEGDFGFGAAFDGDGDRN	A0A0P4VPJ3 JAI57209.1 *Scylla olivacea*	60.9	53.54	6.9%
pp10	63	/	/	/	/	/	6.9%
pp12	48	/	/	/	/	/	6.9%
pp14‐1	40	Arginine kinase	KDFGDVNQFVNVDPDGKFR KLIDDHFLFKE RIISMQMGGDLGQVYRR RVPFSHHDRL	H6VGI3 AFA45340.1 *Scylla paramamosain*	40.3	432.9	58.9%
pp14‐2	40	Tropomyosin	RIQLLEEDLERS	A0A6C0N3G5 QHW05413.1 *Scylla paramamosain*	32.8	35.9	

*Scylla paramamosain*							
Band	MW on SDS‐PAGE (kDa)	Protein name	Identified peptide	Protein accession number and Organism #	Protein MW (kDa)	Score	Sensitization rate
sp3	223	Myosin heavy chain	RYPIYTNRT RDLEETNIQQEAALGLLRK TLNATHPHFIR	A0A8J4XWP0 KAG0710435.1 *Chionoecetes opilio*	274.4	207.7	10.3%
sp4	200	/	/	/	/	/	10.3%
sp6	100	Filamin C	KLPNGHLGISFTPRE KENQFTIDTRD RVHAGGPGLERG RGEQGMPNEFNVWTRE KVYITPSLGEARK KFNGIHIPGSPFRL	A0A5J6X3F8 QFI57017.1 *Scylla paramamosain*	91.1	223.8	10.3%
sp7	95	Glycogen phosphorylase	RDYYFALANTVRD KARPEYMIPVNFYGRV KFFNDGDYIQAVLDRN KVAIQLNDTHPSLAIPELMRI RILVDIEGLTWARA RWPVSMLEHILPRH KLKPLVNDSGFIRT KVIYLENYRV	A0A0P4VYA7 JAI58945.1 *Scylla olivacea*	97.8	488.25	6.9%
sp8	76	Hemocyanin	KHWFSLFNTRQ FALPPGVLEHFETATR YDNNHVEFSFNEGR	A0A223G1C8 AST15994.1 *Scylla serrata*	76.8	125.79	10.3%
sp10	70	Heat shock protein	DAVVTVPAYFNDSQR SINPDEAVAYGAAVQAAILR	A0A1B1FHB2 ANQ44708.1 *Pachygrapsus marmoratus*	74.4	197	6.9%
sp11	65	Phosphoglucomutase	KEGDFGFGAAFDGDGDRN	A0A0P4VPJ3 JAI57209.1 *Scylla olivacea*	60.8	30.2	10.3%
sp17‐1	40	Arginine kinase	KLIDDHFLFKE RIISMQMGGDLGQVYRR	H6VGI3 AFA45340.1 *Scylla paramamosain*	40.3	104.8	44.8%
sp17‐2	40	Tropomyosin	RIQLLEEDLERS	A0A6C0N3G5 QHW05413.1 *Scylla paramamosain*	32.8	35.9	

*Chionoecetes opilio*							
Band	MW on SDS‐PAGE (kDa)	Protein name	Identified peptide	Protein accession number and Organism #	Protein MW (kDa)	Score	Sensitization rate
co2	> 250	/	/	/	/	/	6.9%
co5	150	/	/	/	/	/	6.9%
co9	95	Glycogen phosphorylase	RDYYFALANTVRD KFFNDGDYIQAVLDRN	A0A0P4VYA7 JAI58945.1 *Scylla olivacea*	97.8	488.25	6.9%
co10	76	Hemocyanin	KLLMQELNDHRL KYGGYFPSRPDKV KDFTAEAVITNNNDHEVEATIRV KYDNNHVEFSFNDGRW RWNAIELDRFWTKL	A0A8J4YLL0 KAG0724994.1 *Chionoecetes opilio*	74.8	258.1	24.1%
co13‐1	40	Tropomyosin	KSQLVENELDHAQEQLSAATHKL KAFANAEGEVAALNRR RIQLLEEDLERS KIVELEEELRV KEVDRLEDELVNEKE	A2V735 BAF47267.1 *Chionoecetes opilio*	32.7	328.2	65.5%
co13‐2	40	Arginine kinase	RIISMQMGGDLGQVFRR	A0A1P8D9V4 APU53308.1 *Penaeus japonicus*	40.1 32	132.98	
co14	29	/	/	/	/	/	6.9%

*Eriocheir sinensis*							
Band	MW on SDS‐PAGE (kDa)	Protein name	Identified peptide	Protein accession number and Organism #	Protein MW (kDa)	Score	Sensitization rate
es7	76	Hemocyanin	RKGENFFWVHHQLTVRF KGENFFWVHHQLTVRF KFDLPPGVLEHFETATRD KYDNNGVEYSFNDGRW KYHSGLGLPNRF KYPDNRPHGYPLDRR	K4EJG5 AEG64817.1 *Eriocheir sinensis*	78.3	125.79	10.3%
es9‐1	40	Arginine kinase	RIISMQMGGDLGQVYRR RVPFSHHDRL RGEHTEAEGGIYDISNKR	Q9NH48 AAF43437.1 *Eriocheir sinensis*	40.3	380.8	55.2%
es9‐2	40	Tropomyosin	RIQLLEEDLERS	A0A6C0N3G5 QHW05413.1 *Scylla paramamosain*	32.8	35.9	
es10	29	/	/	/	/	/	10.3%
es11	25	/	/	/	/	/	10.3%

*Paralithodes camtschaticus*							
Band	MW on SDS‐PAGE (kDa)	Protein name	Identified peptide	Protein accession number and Organism #	Protein MW (kDa)	Score	Sensitization rate
pc3	223	Myosin heavy chain	RIEELEQEVEHERQ RDLEETNIQQEAALGLLRK	A0A8J5N6U0 KAG7174238.1 *Homarus americanus*	218.3	125.47	10.3%
pc5	/	/	/	/	/	/	10.3%
pc7/8	76	Hemocyanin	RKGELFFWAHHQLTVRF KFNLPPGVLEHFETATRD	A0A0U1ZZP8 AKC96432.1 *Scylla paramamosain*	74.8	82.16	24.1%
pc9‐1	40	Arginine kinase	RIISMQMGGDLGQVFRR	A0A1P8D9V4 APU53308.1 *Penaeus japonicus*	40.1	132.98	69.0%
pc9‐2	40	Tropomyosin	KALQNAEGEVAALNRR RIQLLEEDLERS KIVELEEELRV	A0A6C0N3G5 QHW05413.1 *Scylla paramamosain*	32.8	91	
pc10	35	Malate dehydrogenase	KDIDAAFLVGAMPRK KNVIIWGNHSSTQFPDVRH RIFGVTTLDIVRA KAGAGSATLSMAYAGARF	A0A8J5CIV7 KAG0711979.1 *Chionoecetes opilio*	35.5	86.24	41.4%
pc11	28	/	/	/	/	/	6.9%

*Note:* MW, Molecular weight of the bands (see Figure 2 for abbreviations) in kilodaltons (kDa); #: protein accession number from UniProt (above) and GenBank (below) database; /: not detected or data unavailable.

### 3.4 Immunoassays for Recombinant MDHs

**FIGURE 3:**
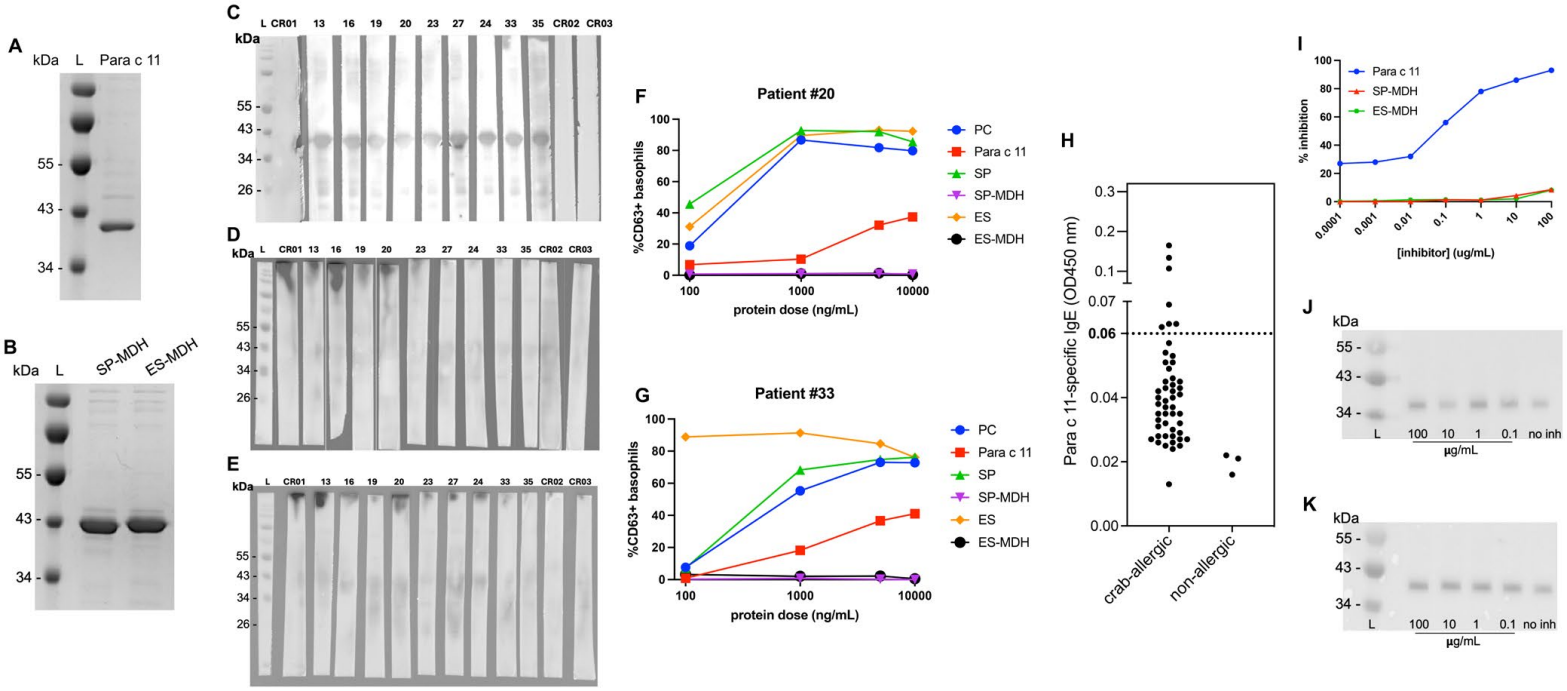
Characterization of recombinant Para c 11. SDS‐PAGE images of (A) Para c 11 and (B) MDH from *S. paramamosain* (SP‐MDH) and *E. sinensis* (ES‐MDH). Immunoblot images of (C) Para c 11, (D) SP‐MDH and (E) ES‐MDH probed with sera from nine crab‐allergic patients and sera from three nonallergic subjects. Results of basophil activation tests from two patients with probable crab allergy, including (F) patient #20 and (G) patient #33 when stimulated with extracts or MDH from *Para. camtschaticus* (PC), *S. paramamosain* (SP) and *E. sinensis* (ES) presented as %CD63+ activated basophils. (H) IgE reactivity of recombinant Para c 11 to sera from 50 crab‐allergic subjects as determined by ELISA. The dotted lines represent cutoff values (OD 450 nm = 0.06), calculated as three times the mean reactivity to sera from nonallergic individuals. The result showed 14% positive IgE binding to Para c 11. (I) Results of inhibition ELISA using serial concentrations of Para c 11, SP‐MDH and ES‐MDH as the inhibitors. Data is presented as % inhibition. Results of inhibition immunoblot against Para c 11 using serial concentrations of (J) SP‐MDH and (K) ES‐MDH as the inhibitors. CR, control; Lane L, molecular weight marker; no inh, no inhibitor.

As shown in Figure 2F and Table 1, MDH exhibited pronounced species‐specific IgE binding in king crab (band PC 10) with a high sensitization rate of 41.4%. To further validate MDH as a king crab‐specific allergen, MDHs of *Para. camtschaticus*, *S. paramamosain*, and *E. sinensis* were cloned and expressed in *E. coli* (Figure 3A,B). Immunoblotting confirmed specific IgE reactivity of rMDH of *Para. camtschaticus* in all allergic patients (*n* = 9; Figure 3C). No IgE reactivity was detected in the three nonallergic controls or against SP‐MDH and ES‐MDH (Figure 3D,E). Functional IgE reactivity of rMDH of *Para. camtschaticus* was also confirmed in two crab‐allergic patients (patients #20 and #33, Table S1) based on BAT that showed maximal basophil activation at 37.4% and 41%, respectively (Figure 3F,G). In contrast, minimal basophil activation was detected for SP‐MDH and ES‐MDH (i.e., maximal activation at 3.2%), despite strong basophil activation when stimulated with the respective crab extracts (up to 93% activation). After that, ELISA was used to measure the reactivity of rMDH of *Para. camtschaticus* against 50 serum samples from crab‐allergic patients (Table S1). rMDH was recognized by IgE in 14% of the tested sera (Figure 3H).

Furthermore, inhibition ELISA and inhibition immunoblot assays were conducted to assess the cross‐reactivity of the rMDHs. In inhibition ELISA, rMDHs from both *S. paramamosain* and *E. sinensis* showed limited IgE inhibition to rMDH of *Para. camtschaticus*, with a maximum of 8.5% and 8.3% inhibition at 100 μg/mL of inhibitors (Figure 3I). On the contrary, rMDH of *Para. camtschaticus* achieved 93.0% inhibition with a clear dose‐dependent inhibitory pattern. Similarly, in inhibition immunoblot, both *S. paramamosain*‐MDH and *E. sinensis* ‐MDH showed minimal IgE inhibition to *Para. camtschaticus*‐MDH at 6.86% and 5.56%, respectively (100 μg/mL of inhibitors; Figure 3J,K). Based on our findings, MDH represents a king crab‐specific allergen and has been registered in the WHO/IUIS Allergen Nomenclature Database as Para c 11.

### 3.5 RNA Expression Level Comparison of Putative Allergens

**FIGURE 4:**
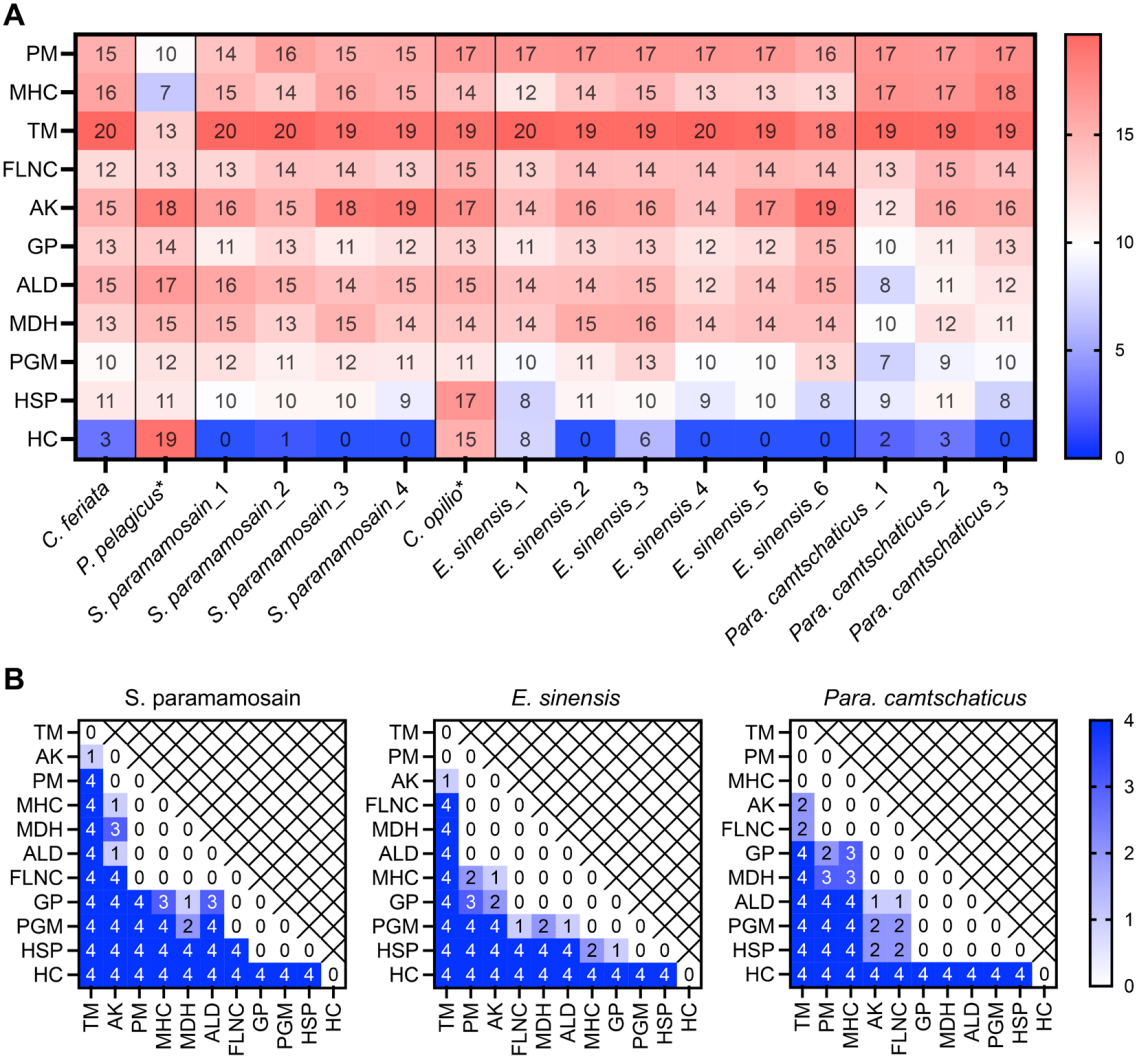
Gene expression comparison. (A) Heatmap comparing the expression levels of allergen candidates across different crab species. Allergen candidates (AK, arginine kinase; ALD, aldolase; filamin C; FLNC; GP, glycogen phosphorylase; HC, hemocyanin; HSP, heat shock protein; MDH, malate dehydrogenase; MHC, myosin heavy chain; PGM, phosphoglucomutase; PM, paramyosin; TM, tropomyosin) in rows; crab species and RNA‐sequencing data analyzed ( *P. pelagicus* SRR8668058; *S. paramamosain*_1, SRR23867489; *S. paramamosain*_2, SRR23867491; *S. paramamosain*_3, SRR23867492; *S. paramamosain*_4 SRR23867493; *E. sinensis* _1, SRR14684335; *E. sinensis* _2, SRR14684346; *E. sinensis* _ 3, SRR14684347; *E. sinensis* _4, SRR7984118; *E. sinensis* _5, SRR7984119; *E. sinensis* _6, SRR7984120; *Para. camtschaticus*_1, ERR5845122; *Para. camtschaticus*_2, ERR5845127; *Para. camtschaticus*_3, SRR10023617) in columns; Note that samples *P. pelagicus* * and *C. opilio* * are from mixed tissues. The numerical values in the cells range from 0 to 20, with a color scale on the right indicating higher expression levels in shades of red and lower expression levels in shades of blue. (B) Heatmaps displaying *p*‐values from individual gene comparisons in species *S. paramamosain*, *E. sinensis* , and *Para. camtschaticus*. The numbers in cells and the legend scale on the right are assigned values as follows: 0, *p* > 0.05; 1, *p* < 0.05; 2, *p* < 0.01; 3, *p* < 0.001; 4, *p* < 0.0001.

To facilitate a comparative assessment of expression profiles for the 11 putative allergens across various crab species, the mapping‐based mode of Salmon was used for RNA sequence data analysis. The heatmap (Figure 4A) depicted the expression levels of different putative allergens across multiple crab species and RNA‐sequencing samples.

TM exhibited elevated expression levels across all examined crab species (values 19–20). Furthermore, the comparative analysis of allergens in *S. paramamosain*, *E. sinensis* , and *Para. camtschaticus* (Figure 4B) provided additional support for the significantly higher expression of TM than the other allergens in these species. Noteworthy exceptions were observed for *P. pelagicus* , with a recorded value of 13 (Figure 4A), wherein the sequencing data encompassed a blend of tissues, including hepatopancreas, gonad, gill, and muscle. In these instances, candidates AK and HC exhibited relatively elevated expression levels in *P. pelagicus* . Alongside TM, the myosin protein PM and MHC also exhibited substantial expression levels throughout the dataset (values 14–18), but not in *P. pelagicus* , where values of 10 and 7 were recorded for PM and MHC, respectively. Moreover, FLNC demonstrated moderate expression levels across all species (values 12–15).

Other than the myosin proteins, the enzyme AK, a known crustacean allergen, consistently displayed high expression levels across all crab species (values over 12), while the candidate GP showed moderate expression levels across all species. Similarly, the enzymes ALD, MDH, and PGM generally exhibited moderate expression levels [10, 11, 12, 13, 14, 15] across the majority of the sequencing samples, with comparatively lower expression observed in one of the three samples (PC_1) in species *Para. camtschaticus* as compared with the other species and putative allergens. Furthermore, HSP generally exhibited low expression levels across most species (values ranging from 8 to 11), except for *C. opilio* , where the sequencing data also originated from a mixture of tissue sources similar to *P. pelagicus* . Conversely, candidate HC was predominantly absent or expressed at low levels across the sequence data, with notable exceptions in *P. pelagicus* and *C. opilio* , where expression values reached 19 and 15, respectively. Overall, excluding the data samples from *P. pelagicus* , *C. opilio* , and *Para. camtschaticus*_1, the expression profiles of 10 allergen genes demonstrated consistency among the species, while HC showed a significantly lower expression level in *S. paramamosain*, *E. sinensis* , and *Para. camtschaticus* (*p* < 0.0001).

### 3.6 Sequence Conservative and Allergenic Epitopes Analysis

**FIGURE 5:**
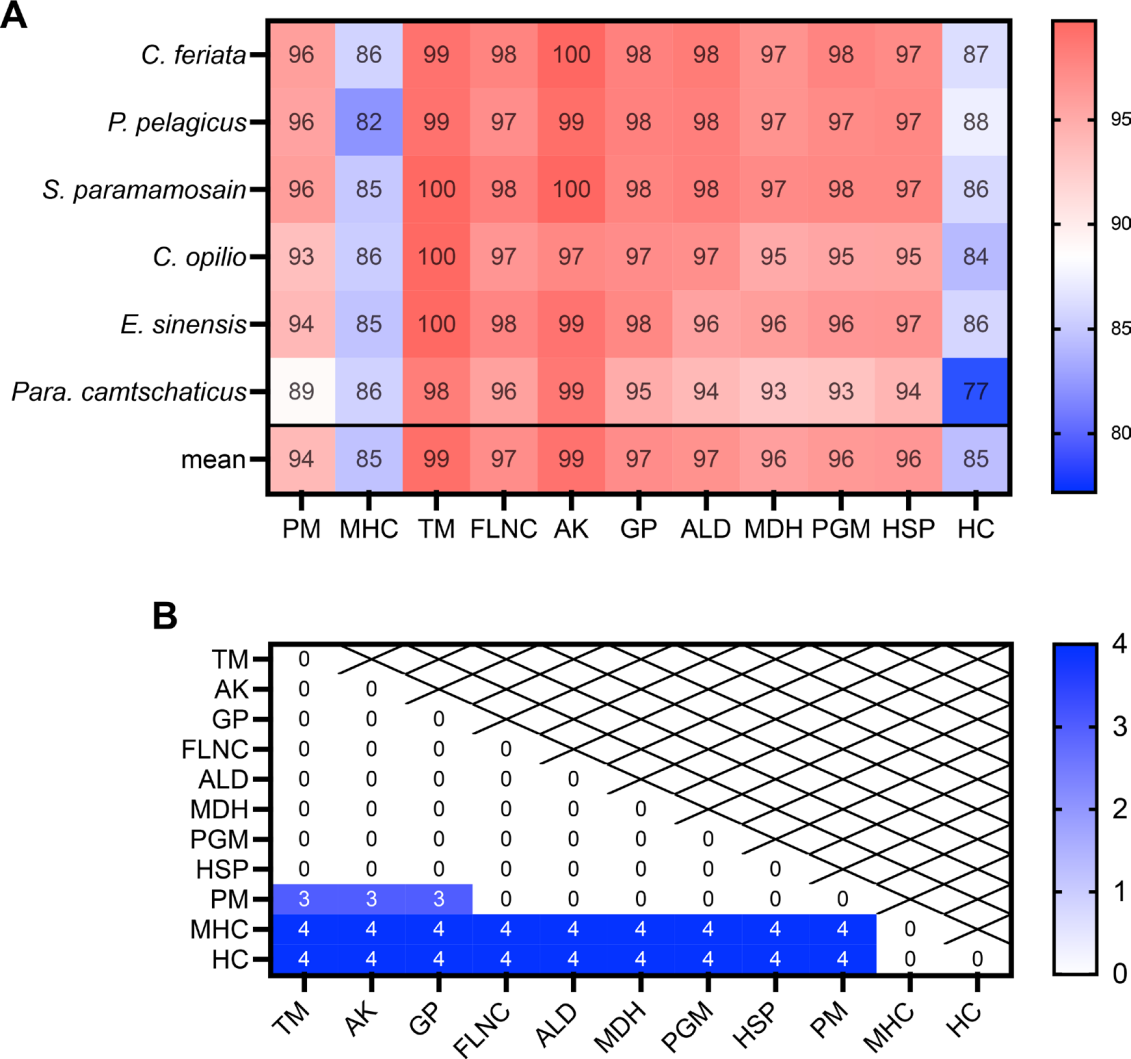
Similarity scores comparison. (A) Heatmaps depicting similarity scores between the consensus sequences of the 11 allergen candidates and their corresponding sequences across the six crab species. Allergen candidates (AK, arginine kinase; ALD, aldolase; filamin C; FLNC; GP, glycogen phosphorylase; HC, hemocyanin; HSP, heat shock protein; MDH, malate dehydrogenase; MHC, myosin heavy chain; PGM, phosphoglucomutase; PM, paramyosin; TM, tropomyosin) in rows and crab species in columns. Cells in the heatmap with the similarity scores colored in red based on the similarity values calculated using the BLOSUM62 scoring matrix. Numerical values in the cells range from 77 to 100, with a color scale on the right indicating higher similarity scores (%) in shades of red and lower in shades of blue. (B) Heatmap displaying *p*‐values from the comparison test between each gene across the species. Numbers in cells and legend scale on the right are assigned values as follows: 3, *p* < 0.001; 4, *p* < 0.0001.

We further analyzed the amino acid sequences of the 11 putative allergens across the six crab species. Diverse alignment techniques were harnessed to pinpoint sequences with the utmost identity to mitigate bias in sequence selection. Sequences from the de novo assembled transcriptomes of claw muscle were preeminent, supplemented by annotated genomes and SRA data. Given the subdued expression levels of HC in muscle tissues, a combination of mixed tissue and whole‐body sequencing data was employed. All protein sequences underwent rigorous scrutiny to ascertain the presence of functional domains supported by robust evidence. Similarity analysis between the consensus sequences of the selected proteins and their corresponding sequences across the species revealed varying degrees of conservation. Multiple sequence alignments for these proteins were generated (Figure S1). The heatmap on the similarity scores calculated using the BLOSUM62 scoring matrix (Figure 5A) demonstrated that all putative allergens exhibited high levels of sequence conservation, with similarity values exceeding 70% among the six species [50]. The columns corresponding to TM, AK, and FLNC exhibited high similarity scores (*p* < 0.001 as compared with other allergens) across all crab species. High similarity scores were also observed for GP (95%–98%), ALD (94%–98%), MDH (93%–97%), PGM (93%–98%), HSP (94%–97%), and PM (89%–96%). MHC and HC displayed lower similarity scores (comparison analysis with *p* < 0.0001). Notably, most proteins originating from the species *Para. camtschaticus* demonstrated lower sequence similarities, with HC in *Para. camtschaticus* showing the most species‐specific variation compared with the five brachyuran species. Analysis by one‐way ANOVA revealed differences among the 11 allergen groups (Figure 5B).

**FIGURE 6:**
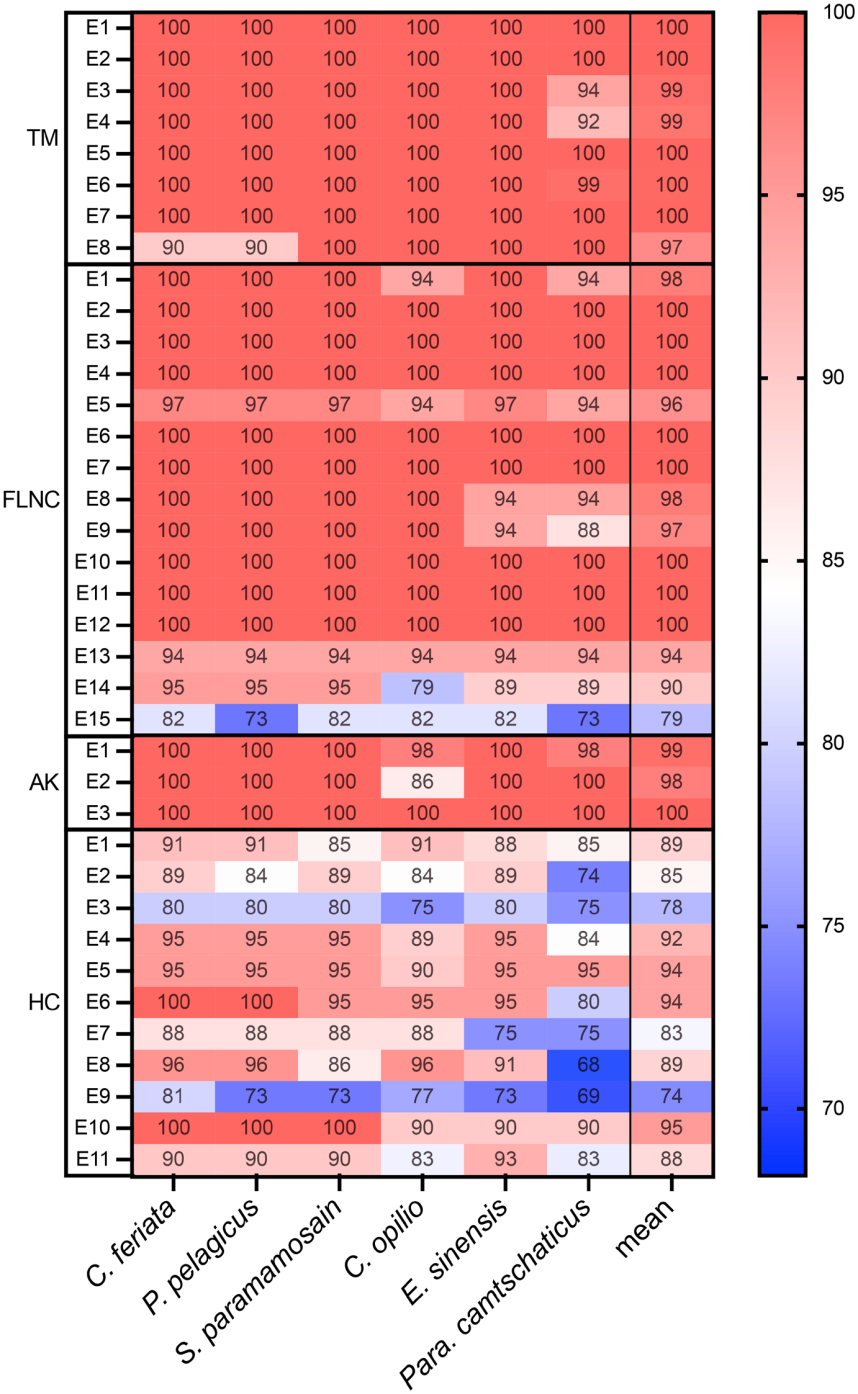
Heatmap depicting the similarity scores between the consensus sequences of the IgE‐binding epitopes in the allergens tropomyosin (TM), arginine kinase (AK) and hemocyanin (HC), and their corresponding sequences in the six crab species. Individual epitopes of allergen candidates (AK, arginine kinase; ALD, aldolase; filamin C; FLNC; GP, glycogen phosphorylase; HC, hemocyanin; HSP, heat shock protein; MDH, malate dehydrogenase; MHC, myosin heavy chain; PGM, phosphoglucomutase; PM, paramyosin; TM, tropomyosin) in rows and crab species in columns. Cells in the heatmap with the similarity scores colored in red based on the similarity values calculated using the BLOSUM62 scoring matrix. Numerical values in the cells range from 68 to 100, with a color scale on the right indicating higher similarity scores (%) in shades of red and lower values in shades of blue.

Furthermore, to analyze the conservation and variability of potential epitope regions of the putative allergens, previously experimentally identified IgE‐binding linear epitopes of four crab allergens were acquired, including IgE‐binding epitopes of TM from *S. paramamosain* [51], FLNC from *S. paramamosain* [52, 53], AK from *S. paramamosain* [18, 54], and HC from *E. sinensis* [55, 56]. The epitope regions of the four allergens (TM: E1‐E8, FLNC: E1‐E15, AK: E1‐E3, and HC: E1‐E11) are listed in Table S3 and illustrated on the sequence alignment (Figure S1C–E,K). These epitope sequences were further compared by similarity analysis calculated with the BLOSUM62 scoring matrix (Figure 6). Our data indicated that all epitopes exhibited a high level of sequence conservation, with similarity values exceeding 68% among the six species. It is evident that most epitopes, particularly those from TM, FLNC, and AK, were located within highly conserved regions, exhibiting similarity scores greater than 90% across the species (Figure 6). On the contrary, the epitopes from HC displayed relatively lower similarity scores, especially in the species *Para. camtschaticus*, which aligned with the results from the analysis of protein sequence similarity.

## 4 Discussion

This study employed traditional protein isolation and immunological assay to comprehensively examine the whole repertoire of crab‐allergenic proteins from six crab species. We identified 11 proteins as putative crab allergens based on our proteomics analysis. Subsequently, transcriptomes of these crabs were acquired for a comparative analysis of the sequences of the identified putative allergens. Our results led to the discovery and registration of a specific king crab allergen, MDH, as Para c 11 in the WHO/IUIS Allergen Nomenclature Database. Proteomic and transcriptomic analyses also revealed a distinct allergen profile in the king crab, highlighting the clinical importance of potential king crab‐specific allergy distinct from allergy to “true crabs”.

Our immunological assay recognized three previously well‐characterized crab allergens, TM, AK, and FLNC. TM and AK were IgE‐positive for all the six crab species, while the IgE reactivity of FLNC was evident in all species except *E. sinensis* and *Para. camtschaticus*. Our results aligned with previous studies regarding the sensitization rates of TM and AK, by which the two major allergens exhibited immunoreactivity against crab‐allergic sera with more than 70% and 50% positive reactions, respectively. For instance, AK exhibited a frequency of 90% reactivity to positive sera in the raw extract of *P. pelagicus* [47]. TM is extensively recognized as the primary allergen responsible for the allergic reactions and cross‐reactivity among different types of shellfish [57], including *C. feriata* [15], *P. pelagicus* [16, 47], *Scylla* spp. [17, 58, 59, 60], *E. sinensis* [48], *C. opilio* [61], and *Para. camtschaticus* [49]. Further, AK, a heat‐sensitive protein, is also recognized as a notable allergenic protein in invertebrates [57], including crabs such as *C. feriata* [62], *P. pelagicus* [47, 62], *Callinectes bellicosus* (Cal b 2) [19], and *Scylla* spp. [18, 58, 60]. Yang et al. also identified FLNC as a novel allergen derived from crabs and crayfish [22, 63]. However, our results indicate a low sensitization rate to FLNC (< 14%) in our study cohort. In addition to immunoblotting, our transcriptomic analysis reveals the consistently high expression level of these three allergens in all six crabs studied, with TM dominating the expression of all allergens, while these allergens also shared a high degree of sequence similarity of > 90% among the crab species. In our study, IgE‐binding protein identification was conducted using immunoblotting with patient sera, which effectively highlights protein bands reactive to IgE but does not precisely pinpoint the specific allergenic proteins within each band. For instance, the 40 kDa band contained both TM and AK, but we could not determine which of these proteins was responsible for IgE binding in individual patients. This limitation should be addressed in future studies using techniques such as IgE inhibition assays or mass spectrometry‐based IgE epitope mapping.

The conservation and variability of potential epitope regions were analyzed by considering previously experimentally identified IgE‐binding linear epitopes from four crab allergens: TM, FLNC, and AK from *S. paramamosain*, and HC from *E. sinensis* . Notably, most epitopes in TM, FLNC, and AK across different crabs are conserved with high sequence similarity (> 80%). This study further confirmed TM and AK as major crab allergens with high molecular cross‐reactivity among the true crabs and king crab. Collectively, this observation facilitates the development of broad‐spectrum diagnostic tools or therapeutic interventions targeting multiple species.

Eight putative novel crab allergens were identified, namely PM, MHC, GP, ALD, MDH, PGM, HSP, and HC based on immunoblotting. Besides TM and AK, other myosin proteins, including PM and MHC, demonstrated high expression levels across most species, further supporting their potential allergenicity. In this investigation, the coding sequences of the 11 candidate allergenic proteins were retrieved across the six species. We noted the significant presence of isoforms in PM, MHC, and TM, underscoring a pronounced functional diversity within motility‐related cytoskeletal proteins in muscle cells. While the intricate landscape of the myosin superfamily isoforms, potentially due to alternative splicing mechanisms, was not exhaustively delved into within this study, these findings highlight the nuanced complexity inherent in these proteins. HC has been recognized as a significant high‐molecular‐weight allergen in the egg of *E. sinensis* , with a notable sensitization frequency of 61% [64]. Additionally, in *Scylla tranquebarica*, HC has been identified as a major allergen [60]. It displayed frequent and robust IgE binding in the raw meat, intestine, and shell extracts of both *Para. camtschaticus* and *Cancer pagurus* [49]. Together with our current findings, the evidence of HC as an allergenic protein in crab species is robust. Previous research conducted by our group also characterized HC as an allergen in the shrimp *Penaeus monodon* , registered as Pen m 7 [30]. Further investigations into the clinical importance of HC cross‐reactivity among different shellfish are highly warranted. Despite being detected as an allergen in all six crab species in the immunoblot assay, transcriptomic analysis shows that HC is either absent or expressed at very low levels in most of the sequence data from muscle, suggesting HC is a migrant protein.

This study also demonstrates MHC, GP, GPM, and HSP as crab putative allergens, representing a novel identification in this research domain. MHC was reported as an allergen in shrimp by immunoblotting and MS [30, 65, 66]. GP was identified by our group as a shrimp allergen in *Penaeus monodon* , known as Pen m 14, with eight of 17 subjects allergic to shrimp exhibiting IgE positivity to recombinant GP [30]. Despite being a minor sensitizing allergen, IgE binding to GP was strongly associated with the manifestation of allergic reactions in the shrimp double‐blind placebo‐controlled food challenge (DBPCFC). GPM is a major meat allergen (beef and lamb) [67] and a major allergen in *Vespa affinis* [68]. Proteins of the Hsp70 family from various sources have been shown as allergens, including mites (Blot 28, Der f 28, Der p 28, and Tyr p 28) [69, 70, 71], sesame seeds [72], pollen [73, 74, 75], insects [68], and fungi (Mal s 10 and Alt a 3) [76, 77]. Moreover, both exogenous and self‐HSP70 play a role in airway inflammatory processes and asthma [78].

As stated earlier, king crabs are not true crabs (infraorder Brachyura) but are close relatives of hermit crabs, both in the infraorder known as Anomura, distinct from Brachyura. Coherently, the dendrogram and SI analysis highlight the distinct clustering of the red king crab *Para. camtschaticus*, indicating substantial divergence from the five true crab species in terms of both protein and allergen profiles. The divergence between the king crab *Para. camtschaticus* and the true crabs is further supported by the lower sequence similarities observed for specific allergens in *Para. camtschaticus* based on transcriptomic analysis, further highlighting species‐specific variations that may affect IgE recognition and binding. Among all crab allergens, HC from *Para. camtschaticus* shows the lowest protein and epitope similarity, and it is noteworthy from the immunoblot assay that serum samples from subjects #1 and #2 reacted only to extracts from the true crabs but not king crabs, as these two subjects reported tolerance to king crab. Remarkably, MDH (Para c 11) was detected as an IgE‐binding protein only in *Para. camtschaticus* and also reactive with sera from subjects who reported allergic reactions to king crab (subjects #3, #4, #16, #20, and #27). These findings underscore the significant importance of crab‐specific allergies, with Para c 11 as a king crab‐specific allergen.

The most important finding from this study is the specific IgE reactivity of MDH from king crab. King crab MDH (Para c 11) showed strong and frequent IgE binding (41.4%) in immunoblot, and IgE reactivity to MDH was not found in “true crab” species. MDH has been reported as an allergen source in fungi (Mala f 4 and Asp f 37) related to atopic dermatitis and asthma [79] and represents a major allergen involved in watermelon allergy [80]. Moreover, Roni Nugraha et al. [81] identified MDH as an allergen in the Pacific oyster using biochemical and computational tools in addition to the antibody reactivity. MDH recombinant protein was assayed for IgE reactivity against 50 crab‐allergic subjects, but yet showed a sensitization rate of only 14%. This can be attributed to the reduced IgE reactivity of recombinant MDH compared with native MDH, and/or the different assay conditions between ELISA and immunoblot. For instance, proteins are heat‐denatured in immunoblot that potentially better expose the linear IgE‐binding epitopes while in ELISA, proteins are immobilized in their native 3D structure. Among the patients tested for recombinant MDH, 7/50 exhibited IgE reactivity. Notably, six of the seven subjects (#24, #30, #31, #32, #34, and #35) had confirmed allergic reactions to king crab, supporting MDH as a species‐specific allergen. It is also noted that MDH from *S. paramamosain*, *C. opilio* , and *E. sinensis* shared 100% amino acid sequence homology while that of king crab to true crabs ranges from 79.2% to 82.4%. Sequence similarity analysis further revealed that king crab MDH had the lowest similarity score (93%) compared with true crabs (95%–97%), reinforcing its identity as a king crab‐specific allergen. To further confirm MDH as a king crab‐specific allergen, MDH homologs from *S. paramamosain* (SP‐MDH) and *E. sinensis* (ES‐MDH) were expressed for immunoblot, BAT, inhibition ELISA, and inhibition immunoblot analysis. No IgE‐binding reactivity and cross‐linking could be detected for SP‐MDH and ES‐MDH. Both homologs also presented minimal inhibition (< 10%) to Para c 11. As a whole, this evidence substantiates MDH as a key and specific allergen in king crab and highlights its potential relevance for species‐specific diagnostic applications. Further characterization of Para c 11 is needed, especially its IgE‐binding epitopes in comparison to other MDH homologs with a larger cohort of patients with challenge‐proven crab allergy.

The patient recruitment relied solely on self‐reported history and their specific IgE level to crab but not oral food challenges to crabs. While our focus was primarily on comparing the allergen repertoire of crabs, this study is limited by the lack of analysis on the heated crab extracts and allergens restricted to the claw muscle without considering the cephalothorax and leg tissues. Future studies can be expanded to include additional tissue sources and also heated extracts to provide a more comprehensive understanding, especially in identifying any neoallergens. Moreover, in our study, the dominant protein with a score threshold > 30 and a matched molecular weight from a single band in immunoblot was identified with MS as the putative crab allergen. Hence, it is possible that additional allergenic proteins within the same band may have been present but not detected due to methodological limitations. Further, in gene expression comparison analysis, tissue‐specific expression biases may exist, particularly in the mixed tissue data from *P. pelagicus* and *C. opilio* . Statistical analyses and consideration of biological replicates may also be necessary to draw robust conclusions. In addition, transcriptomic data alone cannot fully reflect the expression levels of the allergens, and proteomic approaches should be adopted to fully validate the protein content of the major crab allergens. Nevertheless, the identification of allergenic proteins from immunoreactive bands using MS provides valuable insights into the IgE‐binding components of these six crab species and unveils a potential king crab‐specific allergen.

This study represents the first comprehensive analysis of the allergen repertoire of widely consumed crab species, including crucifix crab, blue swimmer crab, green mud crab, snow crab, Chinese mitten crab, and king crab, based on both proteomic and transcriptomic approaches. In addition to known allergens, this study identifies other putative crab allergens and MDH as a novel king crab‐specific allergen that is officially registered as Para c 11. This king crab‐specific allergen Para c 11 and the distinct allergen profile, allergen proteins, and IgE‐binding epitope sequences of king crab highlight the potential of a specific king crab allergy distinct from allergy to true crabs. Such discovery lays the groundwork for enhancing diagnostic approaches and refining management strategies for individuals grappling with crab allergies. Further delineation of Para c 11 and molecular characterization of these crab allergens in a larger cohort of DBPCFC‐diagnosed crab‐allergic patients would aid the development of more accurate predictive models for managing crab allergy and shellfish allergies in general.

## Author Contributions

Conceptualization: K.‐H.C., P.S.C.L., X.X., and C.Y.‐Y.W.; methodology: S.L., J.B., and C.Y.‐Y.W.; data analysis: S.L., J.B., Q.X., and B.S.‐H.W.; investigation: S.L. and J.B.; resources: C.Y.‐Y.W., N.Y.‐H.L., T.‐F.L., K.‐H.C., X.X., S.K.‐W.T., and Q.X.; data curation: S.L., J.B., Q.X., B.S.‐H.W., and C.Y.‐Y.W.; writing – original draft preparation: S.L.; funding acquisition: X.X., C.Y.‐Y.W., and T.‐F.L. All authors read and agreed to the published version of the manuscript.

## Conflicts of Interest

The authors declare no conflicts of interest.

## Supporting information


**Figure S1.** Multiple sequence alignment.

**Table S1.** Clinical characteristics of crab‐allergic patients.

**Table S2.** In‐gel tryptic digest mass spectrometric identification of IgE‐binding proteins isolated from SDS‐PAGE bands.

**Table S3.** Previously identified IgE‐binding linear epitopes of four crab allergens.

## Data Availability

The data that support the findings of this study are available from the corresponding author upon reasonable request.
