# Supplementary material for: Revealing the Diverse Allergenic Protein Repertoire of Six Widely Consumed Crab Species: A Species‐Specific Allergen in King Crab
Source: Allergy. 2025 Jul 30;81(5):1500–21. doi: 10.1111/all.16674 (PMC13139819; doi:10.1111/all.16674)
Supplement: Supplementary file 1 — Figure S1. Multiple sequence alignment. [file ALL-81-1500-s001.pdf]

Figure S1. Multiple sequence alignment

The supplementary pictures below show the amino acid sequence alignments of 11 allergens among six crab species (CF, *Charybdis feriata*; PP, *Portunus pelagicus*; SP, *Scylla paramamosain*; CO, *Chionoecetes opilio*; ES, *Eriocheir sinensis*; PC, *Paralithodes camtschaticus*) for visual comparison of primary structure similarity. (**A**), PM paramyosin; (**B**), myosin heavy chain, (**C**), tropomyosin; (**D**), filamin C; (**E**), arginine kinase; (**F**), glycogen phosphorylase, (**G**), aldolase; (**H**), malate dehydrogenase; (**I**), phosphoglucomutase; (**J**), heat shock protein; (**K**), hemocyanin. The coloured blocks above the sequences indicate the level of sequence conservation, with red representing high conservation (identical amino acid residues) and blue representing low conservation (less than 50% similarity to the consensus sequence). The sequences with bold residues under the blocks are the consensus sequences with a threshold of >50%. Below the consensus sequence is a ruler that indicates the residue positions within the alignment. The amino acids in the individual species sequences that match the consensus sequence are highlighted in yellow. The heatmap at the end of the alignments displays the similarity score between the consensus sequence and the corresponding sequences of each, as depicted in the main text.

A

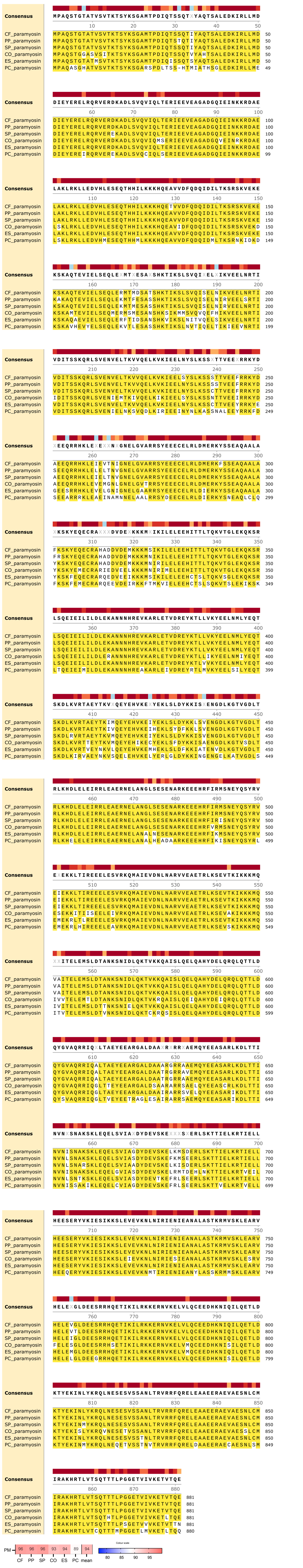
Consensus
CF_paramyosin
PP_paramyosin
SP_paramyosin
CO_paramyosin
ES_paramyosin
PC_paramyosin
PM
CF
PP
SP
CO
ES
PC
mean
Colour scale
80
85
90
95

B

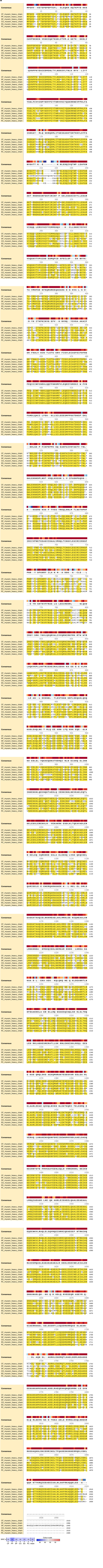

C

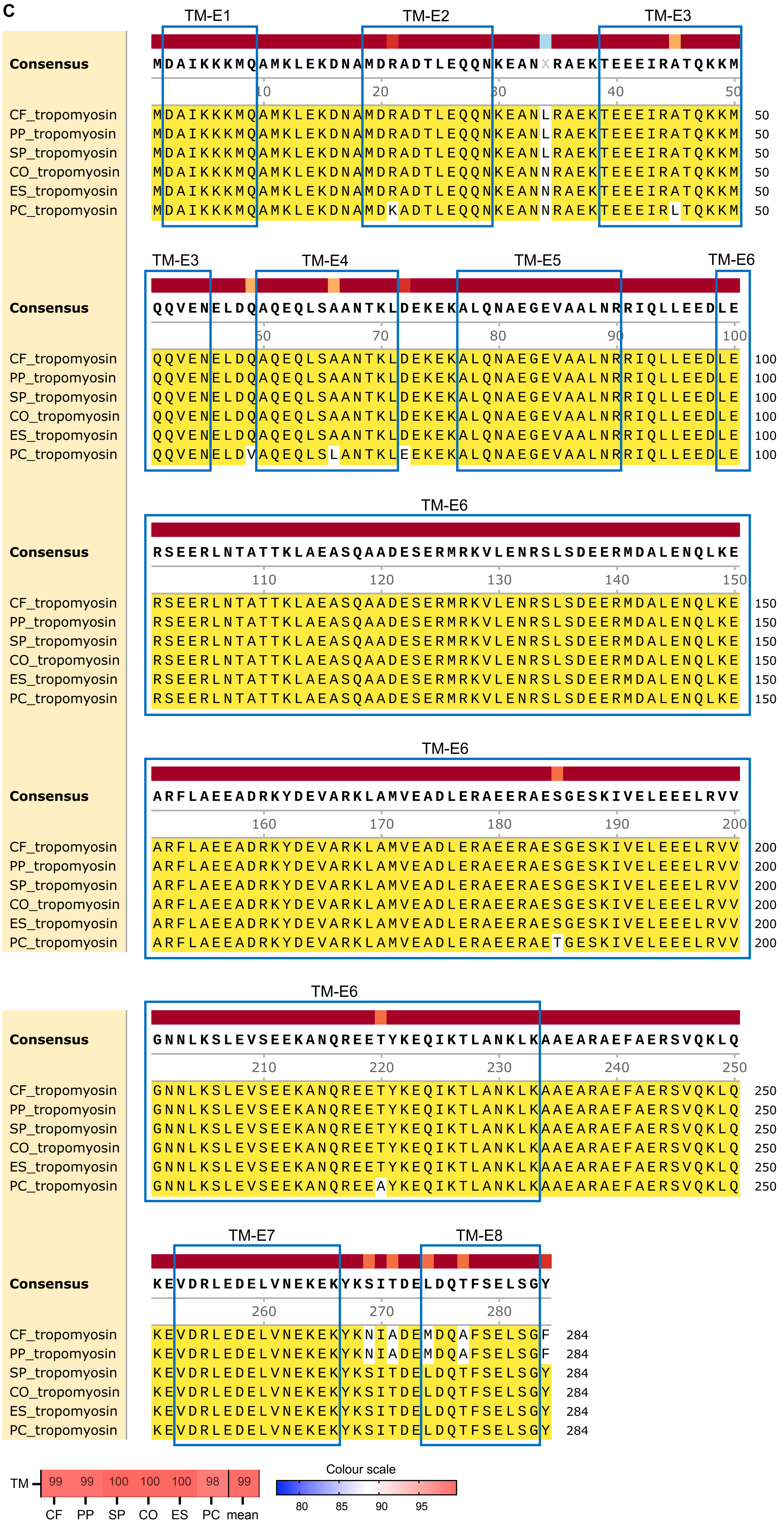

TM-E1
TM-E2
TM-E3
Consensus MDAIKKKMQAMKLEKDNAMDRADTLEQQNKEANXRAEKTEEEIRATQKKM
10 20 30 40 50
CF_tropomyosin MDAIKKKMQAMKLEKDNAMDRADTLEQQNKEANLRAEKTEEEIRATQKKM 50
PP_tropomyosin MDAIKKKMQAMKLEKDNAMDRADTLEQQNKEANLRAEKTEEEIRATQKKM 50
SP_tropomyosin MDAIKKKMQAMKLEKDNAMDRADTLEQQNKEANLRAEKTEEEIRATQKKM 50
CO_tropomyosin MDAIKKKMQAMKLEKDNAMDRADTLEQQNKEANNRAEKTEEEIRATQKKM 50
ES_tropomyosin MDAIKKKMQAMKLEKDNAMDRADTLEQQNKEANNRAEKTEEEIRATQKKM 50
PC_tropomyosin MDAIKKKMQAMKLEKDNAMDKADTLEQQNKEANNRAEKTEEEIRLTQKKM 50
TM-E3
TM-E4
TM-E5
TM-E6
Consensus QQVENELDQAQEQLSAANTKLDEKEKALQNAEGEVAALNRRIQLLEEDLE
60 70 80 90 100
CF_tropomyosin QQVENELDQAQEQLSAANTKLDEKEKALQNAEGEVAALNRRIQLLEEDLE 100
PP_tropomyosin QQVENELDQAQEQLSAANTKLDEKEKALQNAEGEVAALNRRIQLLEEDLE 100
SP_tropomyosin QQVENELDQAQEQLSAANTKLDEKEKALQNAEGEVAALNRRIQLLEEDLE 100
CO_tropomyosin QQVENELDQAQEQLSAANTKLDEKEKALQNAEGEVAALNRRIQLLEEDLE 100
ES_tropomyosin QQVENELDQAQEQLSAANTKLDEKEKALQNAEGEVAALNRRIQLLEEDLE 100
PC_tropomyosin QQVENELDVAQEQLSLANTKLEEKEKALQNAEGEVAALNRRIQLLEEDLE 100
TM-E6
Consensus RSEERLNTATTKLAEASQAADESERMRKVLENRSLSDEERMDALENQLKE
110 120 130 140 150
CF_tropomyosin RSEERLNTATTKLAEASQAADESERMRKVLENRSLSDEERMDALENQLKE 150
PP_tropomyosin RSEERLNTATTKLAEASQAADESERMRKVLENRSLSDEERMDALENQLKE 150
SP_tropomyosin RSEERLNTATTKLAEASQAADESERMRKVLENRSLSDEERMDALENQLKE 150
CO_tropomyosin RSEERLNTATTKLAEASQAADESERMRKVLENRSLSDEERMDALENQLKE 150
ES_tropomyosin RSEERLNTATTKLAEASQAADESERMRKVLENRSLSDEERMDALENQLKE 150
PC_tropomyosin RSEERLNTATTKLAEASQAADESERMRKVLENRSLSDEERMDALENQLKE 150
TM-E6
Consensus ARFLAEEADRKYDEVARKLAMVEADLERAEERAESGESKIVELEEELRVV
160 170 180 190 200
CF_tropomyosin ARFLAEEADRKYDEVARKLAMVEADLERAEERAESGESKIVELEEELRVV 200
PP_tropomyosin ARFLAEEADRKYDEVARKLAMVEADLERAEERAESGESKIVELEEELRVV 200
SP_tropomyosin ARFLAEEADRKYDEVARKLAMVEADLERAEERAESGESKIVELEEELRVV 200
CO_tropomyosin ARFLAEEADRKYDEVARKLAMVEADLERAEERAESGESKIVELEEELRVV 200
ES_tropomyosin ARFLAEEADRKYDEVARKLAMVEADLERAEERAESGESKIVELEEELRVV 200
PC_tropomyosin ARFLAEEADRKYDEVARKLAMVEADLERAEERAETGESKIVELEEELRVV 200
TM-E6
Consensus GNNLKSLEVSEEKANQREETYKEQIKTLANKLKAAEARAEFAERSVQKLQ
210 220 230 240 250
CF_tropomyosin GNNLKSLEVSEEKANQREETYKEQIKTLANKLKAAEARAEFAERSVQKLQ 250
PP_tropomyosin GNNLKSLEVSEEKANQREETYKEQIKTLANKLKAAEARAEFAERSVQKLQ 250
SP_tropomyosin GNNLKSLEVSEEKANQREETYKEQIKTLANKLKAAEARAEFAERSVQKLQ 250
CO_tropomyosin GNNLKSLEVSEEKANQREETYKEQIKTLANKLKAAEARAEFAERSVQKLQ 250
ES_tropomyosin GNNLKSLEVSEEKANQREETYKEQIKTLANKLKAAEARAEFAERSVQKLQ 250
PC_tropomyosin GNNLKSLEVSEEKANQREEAYKEQIKTLANKLKAAEARAEFAERSVQKLQ 250
TM-E7
TM-E8
Consensus KEVDRLEDELVNEKEKYKSITDELDQTFSELSGY
260 270 280
CF_tropomyosin KEVDRLEDELVNEKEKYKNIADEMDQAFSELSGF 284
PP_tropomyosin KEVDRLEDELVNEKEKYKNIADEMDQAFSELSGF 284
SP_tropomyosin KEVDRLEDELVNEKEKYKSITDELDQTFSELSGY 284
CO_tropomyosin KEVDRLEDELVNEKEKYKSITDELDQTFSELSGY 284
ES_tropomyosin KEVDRLEDELVNEKEKYKSITDELDQTFSELSGY 284
PC_tropomyosin KEVDRLEDELVNEKEKYKSITDELDQTFSELSGY 284
TM 99 99 100 100 100 98 99
CF PP SP CO ES PC mean
Colour scale
80 85 90 95

D

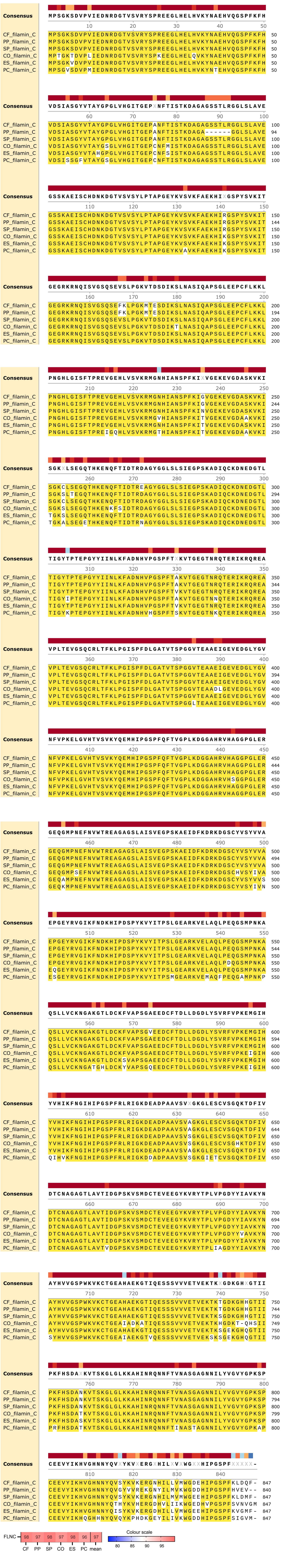
Consensus
CF_filamin_C
PP_filamin_C
SP_filamin_C
CO_filamin_C
ES_filamin_C
PC_filamin_C
FLNC
CF PP SP CO ES PC mean
Colour scale
80 85 90 95

E

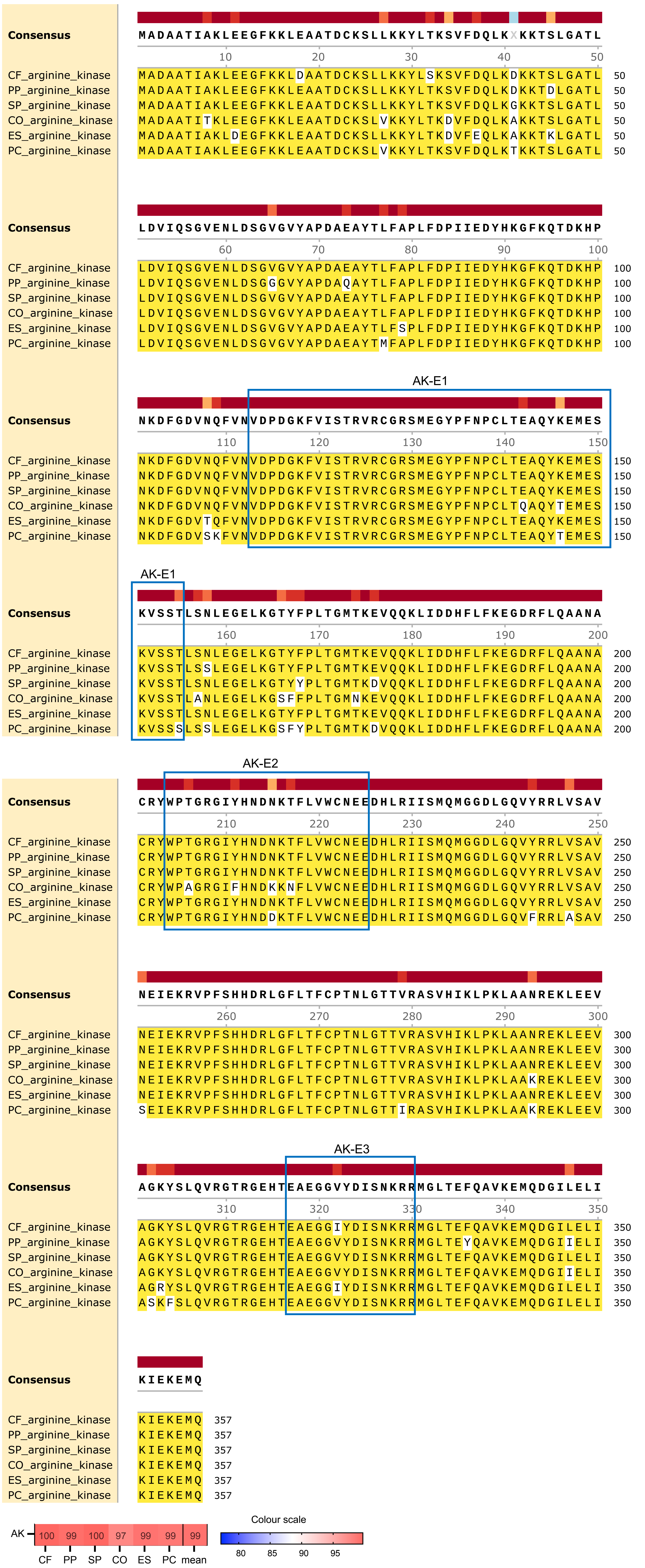
Consensus MADAATIAKLEEGFKKLEAATDCKSLLKKYLTKSVFDQLKXKKTSLGATL
10 20 30 40 50
CF_arginine_kinase MADAATIAKLEEGFKKLDAATDCKSLLKKYLSKSVFDQLKDKKTSLGATL 50
PP_arginine_kinase MADAATIAKLEEGFKKLEAATDCKSLLKKYLTKSVFDQLKDKKTDLGATL 50
SP_arginine_kinase MADAATIAKLEEGFKKLEAATDCKSLLKKYLTKSVFDQLKGKKTSLGATL 50
CO_arginine_kinase MADAATITKLEEGFKKLEAATDCKSLVKKYLTKDVFDQLKAKKTSLGATL 50
ES_arginine_kinase MADAATIAKLDEGFKKLEAATDCKSLLKKYLTKDVFEQLKAKKTKLGATL 50
PC_arginine_kinase MADAATIAKLEEGFKKLEAATDCKSLVKKYLTKSVFDQLKTKKTSLGATL 50
Consensus LDVIQSGVENLDSGVGVYAPDAEAYTLFAPLFDPIIEDYHKGFKQTDKHP
60 70 80 90 100
CF_arginine_kinase LDVIQSGVENLDSGVGVYAPDAEAYTLFAPLFDPIIEDYHKGFKQTDKHP 100
PP_arginine_kinase LDVIQSGVENLDSGGGVYAPDAQAYTLFAPLFDPIIEDYHKGFKQTDKHP 100
SP_arginine_kinase LDVIQSGVENLDSGVGVYAPDAEAYTLFAPLFDPIIEDYHKGFKQTDKHP 100
CO_arginine_kinase LDVIQSGVENLDSGVGVYAPDAEAYTLFAPLFDPIIEDYHKGFKQTDKHP 100
ES_arginine_kinase LDVIQSGVENLDSGVGVYAPDAEAYTLFSPLFDPIIEDYHKGFKQTDKHP 100
PC_arginine_kinase LDVIQSGVENLDSGVGVYAPDAEAYTMFAPLFDPIIEDYHKGFKQTDKHP 100
AK-E1
Consensus NKDFGDVNQFVNVDPDGKFVISTRVRCGRSMEGYPFNPCLTEAQYKEMES
110 120 130 140 150
CF_arginine_kinase NKDFGDVNQFVNVDPDGKFVISTRVRCGRSMEGYPFNPCLTEAQYKEMES 150
PP_arginine_kinase NKDFGDVNQFVNVDPDGKFVISTRVRCGRSMEGYPFNPCLTEAQYKEMES 150
SP_arginine_kinase NKDFGDVNQFVNVDPDGKFVISTRVRCGRSMEGYPFNPCLTEAQYKEMES 150
CO_arginine_kinase NKDFGDVNQFVNVDPDGKFVISTRVRCGRSMEGYPFNPCLTQAQYTEMES 150
ES_arginine_kinase NKDFGDVTQFVNVDPDGKFVISTRVRCGRSMEGYPFNPCLTEAQYKEMES 150
PC_arginine_kinase NKDFGDVSKFVNVDPDGKFVISTRVRCGRSMEGYPFNPCLTEAQYTEMES 150
AK-E1
Consensus KVSSTLSNLEGELKGTYFPLTGMTKEVQQKLIDDHFLFKEGDRFLQAANA
160 170 180 190 200
CF_arginine_kinase KVSSTLSNLEGELKGTYFPLTGMTKEVQQKLIDDHFLFKEGDRFLQAANA 200
PP_arginine_kinase KVSSTLSSLEGELKGTYFPLTGMTKEVQQKLIDDHFLFKEGDRFLQAANA 200
SP_arginine_kinase KVSSTLSNLEGELKGTYYPLTGMTKDVQQKLIDDHFLFKEGDRFLQAANA 200
CO_arginine_kinase KVSSTLANLEGELKGSFFPLTGMNKEVQQKLIDDHFLFKEGDRFLQAANA 200
ES_arginine_kinase KVSSTLSNLEGELKGTYFPLTGMTKEVQQKLIDDHFLFKEGDRFLQAANA 200
PC_arginine_kinase KVSSSLSSLEGELKGSFYPLTGMTKDVQQKLIDDHFLFKEGDRFLQAANA 200
AK-E2
Consensus CRYWPTGRGIYHNDNKTFLVWCNEEDHLRIISMQMGGDLGQVYRRLVSAV
210 220 230 240 250
CF_arginine_kinase CRYWPTGRGIYHNDNKTFLVWCNEEDHLRIISMQMGGDLGQVYRRLVSAV 250
PP_arginine_kinase CRYWPTGRGIYHNDNKTFLVWCNEEDHLRIISMQMGGDLGQVYRRLVSAV 250
SP_arginine_kinase CRYWPTGRGIYHNDNKTFLVWCNEEDHLRIISMQMGGDLGQVYRRLVSAV 250
CO_arginine_kinase CRYWPAGRGIFHNDKKNFLVWCNEEDHLRIISMQMGGDLGQVYRRLVSAV 250
ES_arginine_kinase CRYWPTGRGIYHNDNKTFLVWCNEEDHLRIISMQMGGDLGQVYRRLVSAV 250
PC_arginine_kinase CRYWPTGRGIYHNDDKTFLVWCNEEDHLRIISMQMGGDLGQVFRRLASAV 250
Consensus NEIEKRVPFSHHDRLGFLTFCPTNLGTTVRASVHIKLPKLAANREKLEEV
260 270 280 290 300
CF_arginine_kinase NEIEKRVPFSHHDRLGFLTFCPTNLGTTVRASVHIKLPKLAANREKLEEV 300
PP_arginine_kinase NEIEKRVPFSHHDRLGFLTFCPTNLGTTVRASVHIKLPKLAANREKLEEV 300
SP_arginine_kinase NEIEKRVPFSHHDRLGFLTFCPTNLGTTVRASVHIKLPKLAANREKLEEV 300
CO_arginine_kinase NEIEKRVPFSHHDRLGFLTFCPTNLGTTVRASVHIKLPKLAAKREKLEEV 300
ES_arginine_kinase NEIEKRVPFSHHDRLGFLTFCPTNLGTTVRASVHIKLPKLAANREKLEEV 300
PC_arginine_kinase SEIEKRVPFSHHDRLGFLTFCPTNLGTTIRASVHIKLPKLAAKREKLEEV 300
AK-E3
Consensus AGKYSLQVRGTRGEHTEAEGGVYDISNKRRMGLTEFQAVKEMQDGILELI
310 320 330 340 350
CF_arginine_kinase AGKYSLQVRGTRGEHTEAEGGIYDISNKRRMGLTEFQAVKEMQDGILELI 350
PP_arginine_kinase AGKYSLQVRGTRGEHTEAEGGVYDISNKRRMGLTEYQAVKEMQDGIIELI 350
SP_arginine_kinase AGKYSLQVRGTRGEHTEAEGGVYDISNKRRMGLTEFQAVKEMQDGILELI 350
CO_arginine_kinase AGKYSLQVRGTRGEHTEAEGGVYDISNKRRMGLTEFQAVKEMQDGIIELI 350
ES_arginine_kinase AGRYSLQVRGTRGEHTEAEGGIYDISNKRRMGLTEFQAVKEMQDGILELI 350
PC_arginine_kinase ASKFSLQVRGTRGEHTEAEGGVYDISNKRRMGLTEFQAVKEMQDGILELI 350
Consensus KIEKEMQ
CF_arginine_kinase KIEKEMQ 357
PP_arginine_kinase KIEKEMQ 357
SP_arginine_kinase KIEKEMQ 357
CO_arginine_kinase KIEKEMQ 357
ES_arginine_kinase KIEKEMQ 357
PC_arginine_kinase KIEKEMQ 357
AK 100 99 100 97 99 99 99
CF PP SP CO ES PC mean
Colour scale
80 85 90 95

F

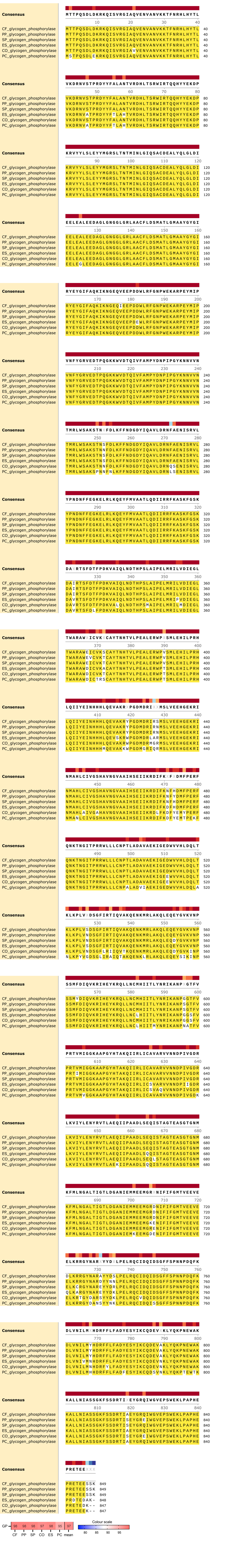
Consensus
CF_glycogen_phosphorylase
PP_glycogen_phosphorylase
SP_glycogen_phosphorylase
ES_glycogen_phosphorylase
CO_glycogen_phosphorylase
PC_glycogen_phosphorylase
GP
CF PP SP CO ES PC mean
Colour scale
80 85 90 95

G

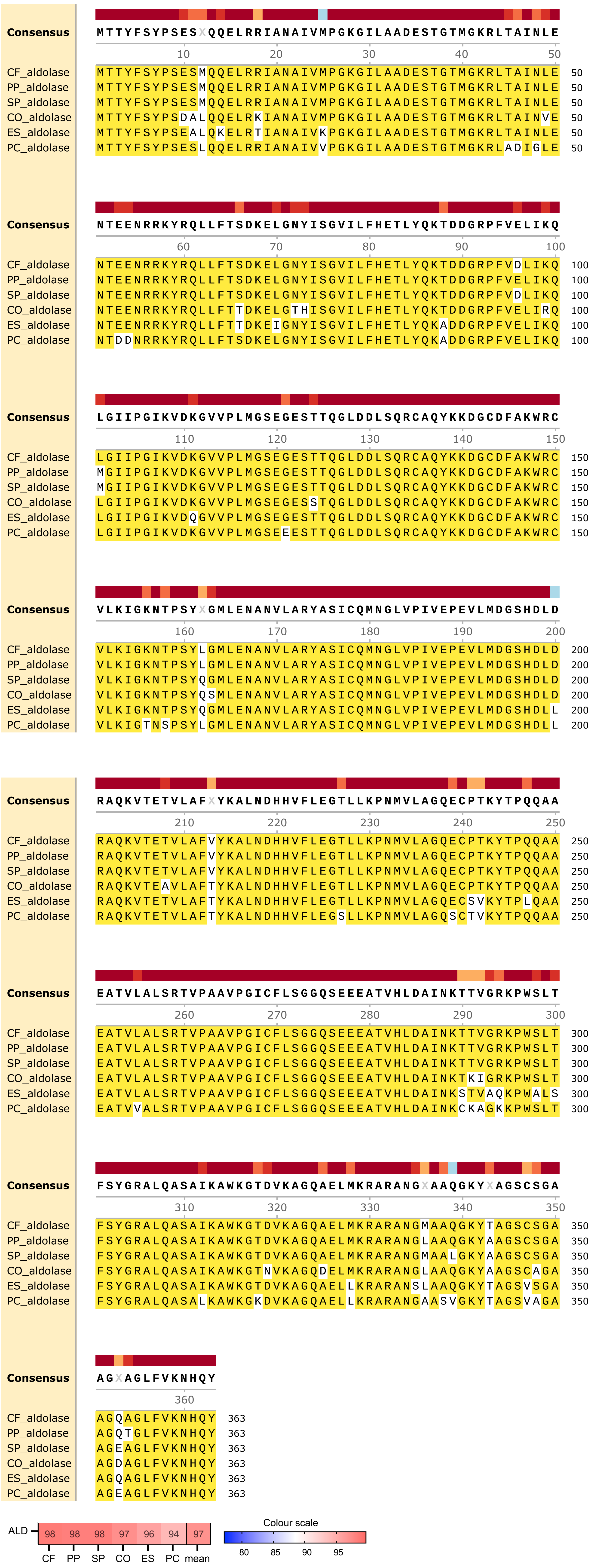

Consensus MTTYFSYPSESXQQELRRIANAIVMPGKGILAADESTGTMGKRLTAINLE
10 20 30 40 50
CF_aldolase MTTYFSYPSESMQQELRRIANAIVMPGKGILAADESTGTMGKRLTAINLE 50
PP_aldolase MTTYFSYPSESMQQELRRIANAIVMPGKGILAADESTGTMGKRLTAINLE 50
SP_aldolase MTTYFSYPSESMQQELRRIANAIVMPGKGILAADESTGTMGKRLTAINLE 50
CO_aldolase MTTYFSYPSDALQQELRKIANAIVMPGKGILAADESTGTMGKRLTAINVE 50
ES_aldolase MTTYFSYPSEALQKELRTIANAIVKPGKGILAADESTGTMGKRLTAINLE 50
PC_aldolase MTTYFSYPSESLQQELRRIANAIVVPGKGILAADESTGTMGKRLADIGLE 50
Consensus NTEENRRKYRQLLFTSDKELGNYISGVILFHETLYQKTDDGRPFVELIKQ
60 70 80 90 100
CF_aldolase NTEENRRKYRQLLFTSDKELGNYISGVILFHETLYQKTDDGRPFVDLIKQ 100
PP_aldolase NTEENRRKYRQLLFTSDKELGNYISGVILFHETLYQKTDDGRPFVELIKQ 100
SP_aldolase NTEENRRKYRQLLFTSDKELGNYISGVILFHETLYQKTDDGRPFVDLIKQ 100
CO_aldolase NTEENRRKYRQLLFTTDKELGTHISGVILFHETLYQKTDDGRPFVELIRQ 100
ES_aldolase NTEENRRKYRQLLFTTDKEIGNYISGVILFHETLYQKADDGRPFVELIKQ 100
PC_aldolase NTDDNRRKYRQLLFTSDKELGNYISGVILFHETLYQKADDGRPFVELIKQ 100
Consensus LGIIPGIKVDKGVVPLMGSEGESTTQGLDDLSQRCAQYKKDGCDFAKWRC
110 120 130 140 150
CF_aldolase LGIIPGIKVDKGVVPLMGSEGESTTQGLDDLSQRCAQYKKDGCDFAKWRC 150
PP_aldolase MGIIPGIKVDKGVVPLMGSEGESTTQGLDDLSQRCAQYKKDGCDFAKWRC 150
SP_aldolase MGIIPGIKVDKGVVPLMGSEGESTTQGLDDLSQRCAQYKKDGCDFAKWRC 150
CO_aldolase LGIIPGIKVDKGVVPLMGSEGESSTQGLDDLSQRCAQYKKDGCDFAKWRC 150
ES_aldolase LGIIPGIKVDQGVVPLMGSEGESTTQGLDDLSQRCAQYKKDGCDFAKWRC 150
PC_aldolase LGIIPGIKVDKGVVPLMGSEEESTTQGLDDLSQRCAQYKKDGCDFAKWRC 150
Consensus VLKIGKNTPSYXGMLENANVLARYASICQMNGLVPIVEPEVLMDGSHDLD
160 170 180 190 200
CF_aldolase VLKIGKNTPSYLGMLENANVLARYASICQMNGLVPIVEPEVLMDGSHDLD 200
PP_aldolase VLKIGKNTPSYLGMLENANVLARYASICQMNGLVPIVEPEVLMDGSHDLD 200
SP_aldolase VLKIGKNTPSYLGMLENANVLARYASICQMNGLVPIVEPEVLMDGSHDLD 200
CO_aldolase VLKIGKNTPSYQSMLENANVLARYASICQMNGLVPIVEPEVLMDGSHDLD 200
ES_aldolase VLKIGKNTPSYQGMLENANVLARYASICQMNGLVPIVEPEVLMDGSHDLL 200
PC_aldolase VLKIGTNSPSYLGMLENANVLARYASICQMNGLVPIVEPEVLMDGSHDLL 200
Consensus RAQKVTETVLAFXYKALNDHHVFLEGTLLKPNMVLAGQECPTKYTPQQAA
210 220 230 240 250
CF_aldolase RAQKVTETVLAFVYKALNDHHVFLEGTLLKPNMVLAGQECPTKYTPQQAA 250
PP_aldolase RAQKVTETVLAFVYKALNDHHVFLEGTLLKPNMVLAGQECPTKYTPQQAA 250
SP_aldolase RAQKVTETVLAFVYKALNDHHVFLEGTLLKPNMVLAGQECPTKYTPQQAA 250
CO_aldolase RAQKVTEAVLAFTYKALNDHHVFLEGTLLKPNMVLAGQECPTKYTPQQAA 250
ES_aldolase RAQKVTETVLAFTYKALNDHHVFLEGTLLKPNMVLAGQECSVKYTPLQAA 250
PC_aldolase RAQKVTETVLAFTYKALNDHHVFLEGSLLKPNMVLAGQSCTVKYTPQQAA 250
Consensus EATVLALSRTVPAAVPGICFLSGGQSEEEATVHLDAINKTTVGRKPWSLT
260 270 280 290 300
CF_aldolase EATVLALSRTVPAAVPGICFLSGGQSEEEATVHLDAINKTTVGRKPWSLT 300
PP_aldolase EATVLALSRTVPAAVPGICFLSGGQSEEEATVHLDAINKTTVGRKPWSLT 300
SP_aldolase EATVLALSRTVPAAVPGICFLSGGQSEEEATVHLDAINKTTVGRKPWSLT 300
CO_aldolase EATVLALSRTVPAAVPGICFLSGGQSEEEATVHLDAINKTKIGRKPWSLT 300
ES_aldolase EATVLALSRTVPAAVPGICFLSGGQSEEEATVHLDAINKSTVAQKPWALS 300
PC_aldolase EATVVALSRTVPAAVPGICFLSGGQSEEEATVHLDAINKCKAGKKPWSLT 300
Consensus FSYGRALQASAIKAWKGTDVKAGQAELMKRARANGXAAQGKYXAGSCSGA
310 320 330 340 350
CF_aldolase FSYGRALQASAIKAWKGTDVKAGQAELMKRARANGMAAQGKYTAGSCSGA 350
PP_aldolase FSYGRALQASAIKAWKGTDVKAGQAELMKRARANGLAAQGKYAAGSCSGA 350
SP_aldolase FSYGRALQASAIKAWKGTDVKAGQAELMKRARANGMAALGKYAAGSCSGA 350
CO_aldolase FSYGRALQASAIKAWKGTNVKAGQDELMKRARANGLAAQGKYAAGSCAGA 350
ES_aldolase FSYGRALQASAIKAWKGTDVKAGQAELLKRARANSLAAQGKYTAGSVSGA 350
PC_aldolase FSYGRALQASALKAWKGKDVKAGQAELLKRARANGAASVGKYTAGSVAGA 350
Consensus AGXAGLFVKNHQY
360
CF_aldolase AGQAGLFVKNHQY 363
PP_aldolase AGQTGLFVKNHQY 363
SP_aldolase AGEAGLFVKNHQY 363
CO_aldolase AGDAGLFVKNHQY 363
ES_aldolase AGQAGLFVKNHQY 363
PC_aldolase AGEAGLFVKNHQY 363
ALD
98 98 98 97 96 94 97
CF PP SP CO ES PC mean
Colour scale
80 85 90 95

H

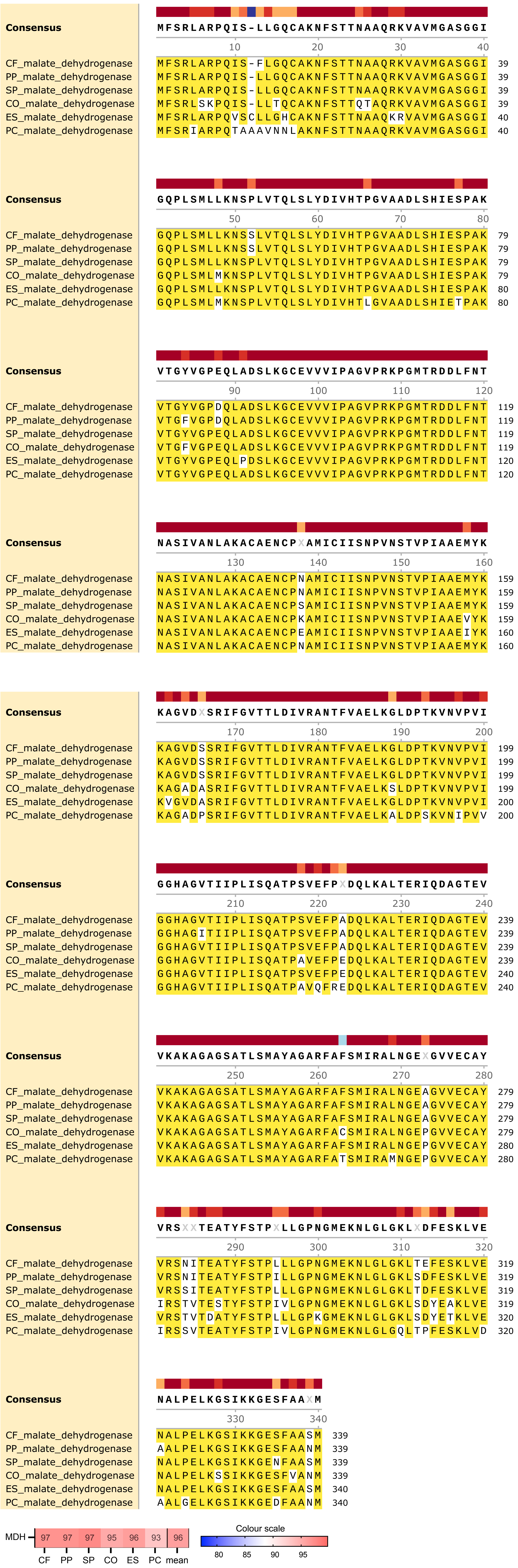

Consensus MFSRLARPQIS-LLGQCAKNFSTTNAAQRKVAVMGASGGI
10 20 30 40
CF_malate_dehydrogenase MFSRLARPQIS-FLGQCAKNFSTTNAAQRKVAVMGASGGI 39
PP_malate_dehydrogenase MFSRLARPQIS-LLGQCAKNFSTTNAAQRKVAVMGASGGI 39
SP_malate_dehydrogenase MFSRLARPQIS-LLGQCAKNFSTTNAAQRKVAVMGASGGI 39
CO_malate_dehydrogenase MFSRLSKPQIS-LLTQCAKNFSTTQTAQRKVAVMGASGGI 39
ES_malate_dehydrogenase MFSRLARPQVSCLLGHCAKNFSTTNAAQKRVAVMGASGGI 40
PC_malate_dehydrogenase MFSRIARPQTAAAVNNLAKNFSTTNAAQRKVAVMGASGGI 40
Consensus GQPLSMLLKNSPLVTQLSLYDIVHTPGVAADLSHIESPAK
50 60 70 80
CF_malate_dehydrogenase GQPLSMLLKNSSLVTQLSLYDIVHTPGVAADLSHIESPAK 79
PP_malate_dehydrogenase GQPLSMLLKNSSLVTQLSLYDIVHTPGVAADLSHIESPAK 79
SP_malate_dehydrogenase GQPLSMLLKNSSLVTQLSLYDIVHTPGVAADLSHIESPAK 79
CO_malate_dehydrogenase GQPLSMLMKNSPLVTQLSLYDIVHTPGVAADLSHIESPAK 79
ES_malate_dehydrogenase GQPLSMLLKNSPLVTQLSLYDIVHTPGVAADLSHIESPAK 80
PC_malate_dehydrogenase GQPLSMLMKNSPLVTQLSLYDIVHTLGVAADLSHIETPAK 80
Consensus VTGYVGPEQLADSLKGCEVVVIPAGVPRKPGMTRDDLFNT
90 100 110 120
CF_malate_dehydrogenase VTGYVGPDQLADSLKGCEVVVIPAGVPRKPGMTRDDLFNT 119
PP_malate_dehydrogenase VTGFVGPDQLADSLKGCEVVVIPAGVPRKPGMTRDDLFNT 119
SP_malate_dehydrogenase VTGYVGPEQLADSLKGCEVVVIPAGVPRKPGMTRDDLFNT 119
CO_malate_dehydrogenase VTGFVGPEQLADSLKGCEVVVIPAGVPRKPGMTRDDLFNT 119
ES_malate_dehydrogenase VTGYVGPEQLPDSLKGCEVVVIPAGVPRKPGMTRDDLFNT 120
PC_malate_dehydrogenase VTGYVGPEQLADSLKGCEVVVIPAGVPRKPGMTRDDLFNT 120
Consensus NASIVANLAKACAENCPXAMICIISNPVNSTVPIAAEMYK
130 140 150 160
CF_malate_dehydrogenase NASIVANLAKACAENCPNAMICIISNPVNSTVPIAAEMYK 159
PP_malate_dehydrogenase NASIVANLAKACAENCPNAMICIISNPVNSTVPIAAEMYK 159
SP_malate_dehydrogenase NASIVANLAKACAENCPSAMICIISNPVNSTVPIAAEMYK 159
CO_malate_dehydrogenase NASIVANLAKACAENCPKAMICIISNPVNSTVPIAAEMYK 159
ES_malate_dehydrogenase NASIVANLAKACAENCPEAMICIISNPVNSTVPIAAEIYK 160
PC_malate_dehydrogenase NASIVANLAKACAENCPNAMICIISNPVNSTVPIAAEMYK 160
Consensus KAGVDXSRIFGVTTLDIVRANTFVAELKGLDPTKVNVPVI
170 180 190 200
CF_malate_dehydrogenase KAGVDSSRIFGVTTLDIVRANTFVAELKGLDPTKVNVPVI 199
PP_malate_dehydrogenase KAGVDSSRIFGVTTLDIVRANTFVAELKGLDPTKVNVPVI 199
SP_malate_dehydrogenase KAGVDSSRIFGVTTLDIVRANTFVAELKGLDPTKVNVPVI 199
CO_malate_dehydrogenase KAGADASRIFGVTTLDIVRANTFVAELKSLDPTKVNVPVI 199
ES_malate_dehydrogenase KVGVDASRIFGVTTLDIVRANTFVAELKGLDPTKVNVPVI 200
PC_malate_dehydrogenase KAGADPSRIFGVTTLDIVRANTFVAELKALDPSKVNIPVV 200
Consensus GGHAGVTIIPLISQATPSVEFPXDQLKALTERIQDAGTEV
210 220 230 240
CF_malate_dehydrogenase GGHAGVTIIPLISQATPSVEFPADQLKALTERIQDAGTEV 239
PP_malate_dehydrogenase GGHAGITIIPLISQATPSVEFPADQLKALTERIQDAGTEV 239
SP_malate_dehydrogenase GGHAGVTIIPLISQATPSVEFPADQLKALTERIQDAGTEV 239
CO_malate_dehydrogenase GGHAGVTIIPLISQATPAVEFPEDQLKALTERIQDAGTEV 239
ES_malate_dehydrogenase GGHAGVTIIPLISQATPSVEFPEDQLKALTERIQDAGTEV 240
PC_malate_dehydrogenase GGHAGVTIIPLISQATPAVQFREDQLKALTERIQDAGTEV 240
Consensus VKAKAGAGSATLSMAYAGARFAFSMIRALNGEXGVVECAY
250 260 270 280
CF_malate_dehydrogenase VKAKAGAGSATLSMAYAGARFAFSMIRALNGEAGVVECAY 279
PP_malate_dehydrogenase VKAKAGAGSATLSMAYAGARFAFSMIRALNGEAGVVECAY 279
SP_malate_dehydrogenase VKAKAGAGSATLSMAYAGARFAFSMIRALNGEAGVVECAY 279
CO_malate_dehydrogenase VKAKAGAGSATLSMAYAGARFACSMIRALNGEPGVVECAY 279
ES_malate_dehydrogenase VKAKAGAGSATLSMAYAGARFAFSMIRALNGEPGVVECAY 280
PC_malate_dehydrogenase VKAKAGAGSATLSMAYAGARFATSMIRAMNGEPGVVECAY 280
Consensus VRSXXTEATYFSTPXLLGPNGMEKNLGLGKLXDFESKLVE
290 300 310 320
CF_malate_dehydrogenase VRSNITEATYFSTPLLLGPNGMEKNLGLGKLTEFESKLVE 319
PP_malate_dehydrogenase VRSNITEATYFSTPLLLGPNGMEKNLGLGKLSDFESKLVE 319
SP_malate_dehydrogenase VRSSITEATYFSTPLLLGPNGMEKNLGLGKLTDFESKLVE 319
CO_malate_dehydrogenase IRSTVTESTYFSTPIVLGPNGMEKNLGLGKLSDYEAKLVE 319
ES_malate_dehydrogenase VRSTVTDATYFSTPLLLGPKGMEKNLGLGKLSDYETKLVE 320
PC_malate_dehydrogenase IRSSVTEATYFSTPIVLGPNGMEKNLGLGQLTPFESKLVD 320
Consensus NALPELKGSIKKGESFAAXM
330 340
CF_malate_dehydrogenase NALPELKGSIKKGESFAASM 339
PP_malate_dehydrogenase AALPELKGSIKKGESFAANM 339
SP_malate_dehydrogenase NALPELKGSIKKGENFAASM 339
CO_malate_dehydrogenase NALPELKSSIKKGESFVANM 339
ES_malate_dehydrogenase NALPELKGSIKKGESFAASM 340
PC_malate_dehydrogenase AALGELKGSIKKGEDFAANM 340
MDH 97 97 97 95 96 93 96
CF PP SP CO ES PC mean
Colour scale
80 85 90 95

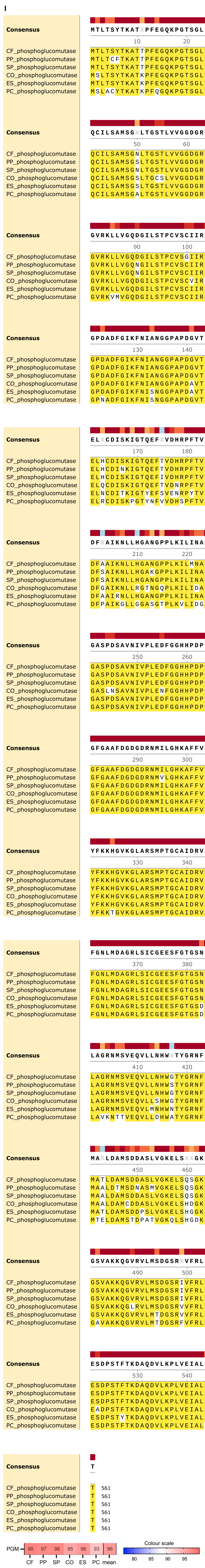
l
Consensus
MTLTSYTKATxPFEGQKPGTSGLRKAVKVFEKEHYTENFV
QCILSAMSGxLTGSTLVVGGDGRYFGKKAVDIIIKMCAAN
GVRKLLVGQDGILSTPCVSCIIRKHQTAGGIVLTASHNPG
GPDADFGIKFNIANGGPAPDGVTNKIYEISKNIVEYKICP
ELxCDISKIGTQEFxVDHRPFTVEVVDSVxDYVxYMKEIF
DFxAIKNLLHGANGPPLKILINAMHGVTGPYVRRIFLEEL
GASPDSAVNIVPLEDFGGHHPDPNLTYAADLVKAMKEGDF
GFGAAFDGDGDRNMILGHKAFFVTPSDSVAVIADNTDYIP
YFKKHGVKGLARSMPTGCAIDRVAQKKGVEVFEVPTGWKY
FGNLMDAGRLSICGEESFGTGSNHIREKDGIWAVLAWLSI
LAGRNMSVEQVLLNHWxTYGRNFFTRYDYENCESAPCNKM
MAxLDAMSDDASLVGKELSxxGKTYTIAKIDNFAYTDPID
GSVAKKQGVRVLMSDGSRxVFRLSGTGSSGATVRMYVESY
ESDPSTFTKDAQDVLKPLVEIALSLAKKEYTGRDKPTVI
T
CF_phosphoglucomutase
PP_phosphoglucomutase
SP_phosphoglucomutase
CO_phosphoglucomutase
ES_phosphoglucomutase
PC_phosphoglucomutase
PGM
98 97 98 95 96 93 96
CF PP SP CO ES PC mean
Colour scale
80 85 90 95

J

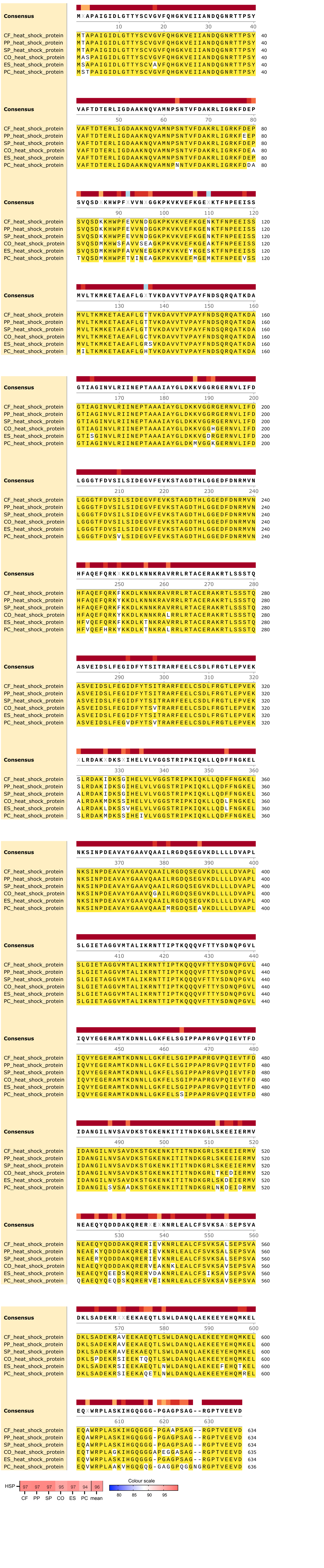
Consensus
CF_heat_shock_protein
PP_heat_shock_protein
SP_heat_shock_protein
CO_heat_shock_protein
ES_heat_shock_protein
PC_heat_shock_protein
HSP
CF PP SP CO ES PC mean
Colour scale
80 85 90 95

K

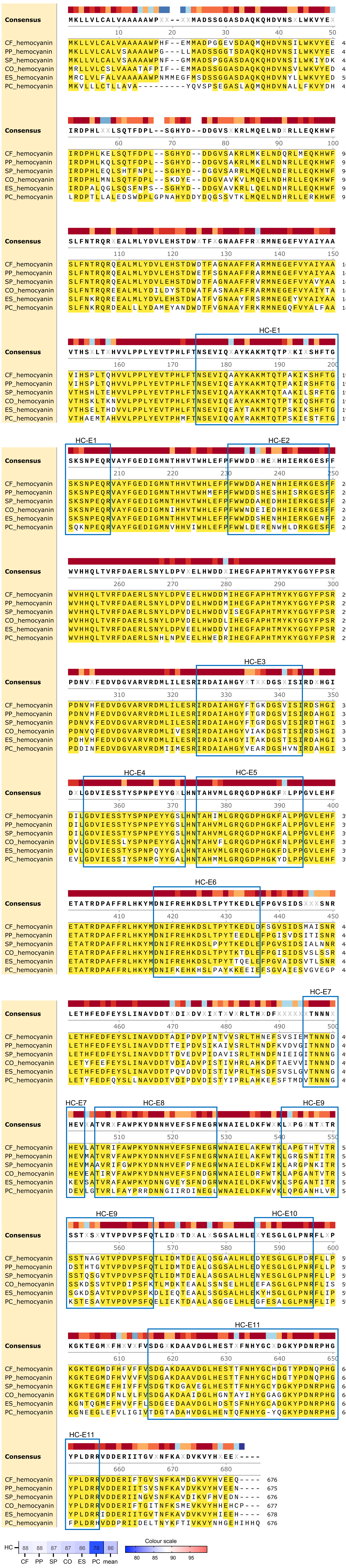
Consensus
CF_hemocyanin
PP_hemocyanin
SP_hemocyanin
CO_hemocyanin
ES_hemocyanin
PC_hemocyanin
HC-E1
HC-E2
HC-E3
HC-E4
HC-E5
HC-E6
HC-E7
HC-E8
HC-E9
HC-E10
HC-E11
HC
CF
PP
SP
CO
ES
PC
mean
Colour scale
