# Supplementary material for: Revealing the Diverse Allergenic Protein Repertoire of Six Widely Consumed Crab Species: A Species‐Specific Allergen in King Crab
Source: Allergy. 2025 Jul 30;81(5):1500–21. doi: 10.1111/all.16674 (PMC13139819; doi:10.1111/all.16674)
Supplement: Supplementary file 3 — Table S2. In‐gel tryptic digest mass spectrometric identification of IgE‐binding proteins isolated from SDS‐PAGE bands. [file ALL-81-1500-s003.docx]

## Table S2. In-gel tryptic digest mass spectrometric identification of IgE binding proteins isolated from SDS-PAGE bands and their sensitization rate in WB. #: Protein accession number from UniProt (above) and GenBank (below) database; /: Not detected or data unavailable

#### **Table S2 -1** Charybdis feriata

| Band | MW on SDS-PAGE (kDa) | Protein name | Identified peptide | Protein Accession number and Organism # | Protein MW  (kDa) | Score | Reactive serum | Sensitization rate |
| --- | --- | --- | --- | --- | --- | --- | --- | --- |
| cf1 | >250 | paramyosin | RQAISLQEIQAHYDEIQRQ  RQLQTTLDQYGVAQRR | A0A8J4YAP8  KAG0719394.1  *Chionoecetes opilio* | 102.2 | 60.57 | 4, 5 | 6.9% |
| cf2 | >250 | twitchin like 1 | KQTILDIPFAQRT  KGTVPNLTEGQEYEFRI  RLLEGTQYEFR  RVINLTEHQEYEFRA | A0A8J5N9Y3  KAG7176305.1  *Homarus americanus* | 854.7 | 129.3 | 5 | 3.4% |
| cf3 | 223 | myosin heavy chain | RYPIYTNRT  RNYATELFRS  RGQLELSQVRQ | A0A8J4XWP0  KAG0710435.1  *Chionoecetes opilio* | 165.8 | 142.7 | 4, 5, 8,14,17 | 17.2% |
| cf4 | 200 | paramyosin | RLKHDLELEIRR  RQAISLQEIQAHYDEIQRQ  RQLQTTLDQYGVAQRR | A0A8J4YAP8  KAG0719394.1  *Chionoecetes opilio* | 102.2 | 105.6 | 5, 7 | 6.9% |
| cf5 | 150 | muscle M-line assembly protein | RVIADPKPTITWYRD  RVTATVTLEKEELYRI  KYVVQMEMIRA | A0A5B7CW41  MPC14057.1  *Portunus trituberculatus* | 97.8 | 95.5 | 4 | 3.4% |
| cf6 | 115 | alpha-actinin | KGISQEQLNEFRS  RTINEVENQILTRD  RVGWEQLLTSINRT  RILAGDKPYILPEELQRE | A0A8J4YHK7  KAG0726703.1  *Chionoecetes opilio* | 103.2 | 126.4 | 4 | 3.4% |
| cf7 | 100 | filamin C | KKLPNGHLGISFTPRE  RVHAGGPGLERG  KVYITPSLGEARK  KFNGIHIPGSPFRL | A0A5J6X3F8  QFI57017.1  *Scylla paramamosain* | 91.1 | 155.9 | 2 ,4, 12, 17 | 13.8% |
| cf8/9 | 76 | hemocyanin | KGESFFWVHHQLTVR  FALPPGVLEHFETATR  YDNNHVEFSFNEGR | A0A0U1ZZD3  AKC96430.1  *Scylla paramamosain* | 77.8 | 211 | 1, 2, 5, 7, 10, 12, 14 | 24.1% |
| cf10 | 70 | heat shock protein | DAVVTVPAYFNDSQR  SINPDEAVAYGAAVQAAILR  MVNHFAQEFQR | A0A8J4YVP5  KAG0727865.1  *Chionoecetes opilio* | 70 | 172.3 | 1, 4 | 6.9% |
| cf11 | 65 | glucose-6-phosphate isomerase | KNWFLDTAKDPAAVAK  KVFEGNRPTNSIMVERI | A0A5B7DVE4  MPC25057.1  *Portunus trituberculatus* | 63.4 | 86.7 | 4 | 3.4% |
| cf12 | 63 | / | / | / | / | / | 1 | 3.4% |
| cf13 | 60 | titin | RAGPVPPSFDQTIQDTQSNPGNLVRL | A0A5B7DEM6  MPC19546.1  *Portunus trituberculatus* | 74.1 | 86.34 | 4 | 3.4% |
| cf14 | 48 | enolase | RGNPTVEVDLYTQKG  RAAVPSGASTGVHEALEMRD  KLAMQEFMILPTGATSFTEAMRM  KIEIGMDVAASEFYKG  KVNQIGSVTESIDAHLLAKK | A0A8J4XSY7  KAG0711044.1  *Chionoecetes opilio* | 47.1 | 369.2 | 1 | 3.4% |
| cf15 | 44 | phosphoglycerate kinase | RKLGDVYINDAFGTAHRA | A0A0P4WMP5  JAI67940.1  *Scylla olivacea* | 44.6 | 139.8 | 2 | 3.4% |
| cf16 | 43 | beta-actin | RAVFPSIVGRPRH  KIWHHSFYNELRV  KSYELPDGQVITIGNERF  KDLFANNVLSGGTTMYPGIADRM  KEEYDESGPGIVHRK | H9B3Y9  AFC88033.1  *Scylla paramamosain* | 41.8 | 373.2 | 1 | 3.4% |
| cf17 | 42 | aldolase | KGVVPLMGSEGESTTQGLDDLSQRC  KYTPQQAAEATVLALSRT  RKPWSLTFSYGRA  KNTPSYQGMLENANVLARY | A0A8J5BSY0  KAG0703349.1  *Chionoecetes opilio* | 39.6 | 294.4 | 1, 2 | 6.9% |
| cf18-1 | 40 | arginine kinase | KLIDDHFLFKE  RIISMQMGGDLGQVYRR  RLVSAVNEIEKRV  RVPFSHHDRL  RGEHTEAEGGIYDISNKR | Q9NH48  AAF43437.1  *Eriocheir sinensis* | 43.4 | 404 | 3, 4, 7, 9, 10, 11, 12, 13, 14, 16, 17, 19, 20, 21, 22, 23, 25, 27, 28, 29 | 69.0% |
| cf18-2 | 40 | tropomyosin | RIQLLEEDLERS | A0A6C0N3G5  QHW05413.1  *Scylla paramamosain* | 32.8 | 35.9 |  |  |
| cf19 | 36 | glyceraldehyde-3-phosphate dehydrogenase | KAGAEYVVESTGVFTTIEKA  KVVSWYDNEFGYSNRV | A0A5B7G3T3  AFQ60506.1  *Portunus trituberculatus* | 35.7 | 190.7 | 1 | 3.4% |
| cf20 | 35 | / | / | / | / | / | 1 | 3.4% |
| cf21 | 28 | / | / | / | / | / | 1, 17 | 6.9% |
| cf22 | 22 | / | / | / | / | / | 1 | 3.4% |
| cf23-1 | 20 | calponin | KQVSQVSTTLFALGRA | A0A0P4VWA4  JAI57033.1  *Scylla olivacea* | 20.4 | 67.03 | 4 | 3.4% |
| cf23-2 | 20 | muscle-specific protein 20 | KYGVPHEEIFQTADLFERR | A0A8J4Y207  KAG0718948.1  *Chionoecetes opilio* | 26.1 | 44.69 |  |  |

#### **Table S2-2** Portunus pelagicus

| Band | MW on SDS-PAGE (kDa) | Protein name | Identified peptide | Protein Accession number and Organism # | Protein MW  (kDa) | Score | Reactive serum | Sensitization rate |
| --- | --- | --- | --- | --- | --- | --- | --- | --- |
| pp1 | >250 | paramyosin | RQAISLQEIQAHYDEIQRQ  RQLQTTLDQYGVAQRR | A0A8J4YAP8  KAG0719394.1  *Chionoecetes opilio* | 102.2 | 176.7 | 5 | 3.4% |
| pp2 | >250 | / | / | / | / | / | 4 | 3.4% |
| pp3 | 223 | myosin heavy chain | TLNATHPHFIRC  RDLEEINIQHEAALSQLRK | A0A5B7DQZ0  KAG0710435.1  *Chionoecetes opilio* | 218.8 | 68.6 | 1, 5 | 6.9% |
| pp4 | 110 | calcium-transporting ATPase | REIVPGDVVEVSVGDKIPADLRL  KEFTLEFSRD | A0A6A4VBB0  KAF0287632.1  *Amphibalanus amphitrite* | 112.3 | 52.5 | 1 | 3.4% |
| pp5 | 100 | filamin C | KKLPNGHLGISFTPRE  KVYITPSLGEARK  KFNGIHIPGSPFRL | A0A5J6X3F8  QFI57017.1  *Scylla paramamosain* | 90.9 | 148.7 | 2, 4, 14, 17 | 13.8% |
| pp6 | 95 | glycogen phosphorylase | KARPEYMIPVNFYGRV  KFFNDGDYIQAVLDRN  RILVDIEGLTWARA  KLKPLVNDSGFIRT  KVIYLENYRV  KLPAPHEPRE | A0A0P4VYA7  JAI58945.1  *Scylla olivacea* | 97.8 | 210.1 | 1, 5 | 6.9% |
| pp7/8 | 76 | hemocyanin | KGESFFWVHHQLTVR  FALPPGVLEHFETATR  YDNNHVEFSFNEGR | A0A0U1ZZD3  AKC96430.1  *Scylla paramamosain* | 77.8 | 141.6 | 1, 4, 5 | 10.3% |
| pp9 | 65 | phosphoglucomutase | KEGDFGFGAAFDGDGDRN | A0A0P4VPJ3  JAI57209.1  *Scylla olivacea* | 60.9 | 53.54 | 1, 4 | 6.9% |
| pp10 | 63 | / | / | / | / | / | 1, 5 | 6.9% |
| pp11 | 60 | titin | REIFLEDSGVFAVVAENRG  RAGPVPPSFDQTIQDTQSNPGNLVRL | A0A5B7DEM6  MPC19546.1  *Portunus trituberculatus* | 98.3 | 183.9 | 4 | 3.4% |
| pp12 | 48 | / | / | / | / | / | 1, 5 | 6.9% |
| pp13 | 42 | aldolase | KKPWALTFSYGRA  KNTPSYQGMLENANVLARY | A0A068FCL9  AID61753.1  *Penaeus chinensis* | 39.9 | 137.3 | 1 | 3.4% |
| pp14-1 | 40 | arginine kinase | KDFGDVNQFVNVDPDGKFR  KLIDDHFLFKE  RIISMQMGGDLGQVYRR  RVPFSHHDRL | H6VGI3  AFA45340.1  *Scylla paramamosain* | 40.3 | 432.9 | 3, 4, 9, 10, 11,12, 13, 14, 16, 17,19, 20, 21, 22, 23, 25, 27 | 58.9% |
| pp14-2 | 40 | tropomyosin | RIQLLEEDLERS | A0A6C0N3G5  QHW05413.1  *Scylla paramamosain* | 32.8 | 35.9 |  |  |
| pp15 | 36 | glyceraldehyde-3-phosphate dehydrogenase | KAGAEYVVESTGVFTTIEKA  KVVSWYDNEFGYSNRV  KIIVYNEMKPENIPWSKA | J7K2U9  AFQ60506.1  *Scylla olivacea* | 38.6 | 202.8 | 1 | 3.4% |
| pp16-1 | 20 | muscle-specific protein 20 | KYGVPHEEIFQTADLFERR | A0A8J4Y207  KAG0718948.1  *Chionoecetes opilio* | 17.8 | 128.1 | 4 | 3.4% |
| pp16-2 | 20 | troponin C | KGYITPETVGTILRM | A0A7U1BGE6  QQY98960.1  *Scylla paramamosain* | 16.8 | 43.6 |  |  |
| pp16-3 | 20 | calponin | KQVSQVSTTLFALGRA | A0A0P4VWA4  JAI57033.1  *Scylla olivacea* | 20.4 | 43.5 |  |  |

#### **Table S2 -3** Scylla paramamosain

| Band | MW on SDS-PAGE (kDa) | Protein name | Identified peptide | Protein Accession number and Organism # | Protein MW  (kDa) | Score | Reactive serum | Sensitization rate |
| --- | --- | --- | --- | --- | --- | --- | --- | --- |
| sp1 | >250 | / | / | / | / | / | 4 | 3.4% |
| sp2 | >250 | twitchin like 1 | KQTILDIPFAQRT  KGTVPNLTEGQEYEFRI  RLLEGTQYEFRV  RVINLTEHQEYEFRA | A0A8J5N9Y3  KAG7176305.1  *Homarus americanus* | / | 109.3 | 5 | 3.4% |
| sp3 | 223 | myosin heavy chain | RYPIYTNRT  RDLEETNIQQEAALGLLRK  TLNATHPHFIR | A0A8J4XWP0  KAG0710435.1  *Chionoecetes opilio* | 274.4 | 207.7 | 1, 5, 7 | 10.3% |
| sp4 | 200 | / | / | / | / | / | 4, 5, 7 | 10.3% |
| sp5 | 150 | muscle M-line assembly protein | RVIADPKPTITWYRD | A0A5B7CW41  MPC14057.1  *Portunus trituberculatus* | / | 30.5 | 4 | 3.4% |
| sp6 | 100 | filamin C | KLPNGHLGISFTPRE  KENQFTIDTRD  RVHAGGPGLERG  RGEQGMPNEFNVWTRE  KVYITPSLGEARK  KFNGIHIPGSPFRL | A0A5J6X3F8  QFI57017.1  *Scylla paramamosain* | 91.1 | 223.8 | 2, 4, 17 | 10.3% |
| sp7 | 95 | glycogen phosphorylase | RDYYFALANTVRD  KARPEYMIPVNFYGRV  KFFNDGDYIQAVLDRN  KVAIQLNDTHPSLAIPELMRI  RILVDIEGLTWARA  RWPVSMLEHILPRH  KLKPLVNDSGFIRT  KVIYLENYRV | A0A0P4VYA7  JAI58945.1  *Scylla olivacea* | 97.8 | 488.25 | 4, 5 | 6.9% |
| sp8/9 | 76 | hemocyanin | KHWFSLFNTRQ FALPPGVLEHFETATR  YDNNHVEFSFNEGR | A0A223G1C8  AST15994.1  *Scylla serrata* | 76.8 | 125.79 | 1, 5, 7 | 10.3% |
| sp10 | 70 | heat shock protein | DAVVTVPAYFNDSQR  SINPDEAVAYGAAVQAAILR | A0A1B1FHB2  ANQ44708.1  *Pachygrapsus marmoratus* | 74.4 | 197 | 4, 5 | 6.9% |
| sp11 | 65 | phosphoglucomutase | KEGDFGFGAAFDGDGDRN | A0A0P4VPJ3  JAI57209.1  *Scylla olivacea* | 60.8 | 30.2 | 1, 4, 7 | 10.3% |
| sp12 | 63 | pyruvate kinase | KNIDSIIEESDGIMIARG | A0A0P4WF29  JAI59679.1  *Scylla olivacea* | 57.2 | 52.69 | 1 | 3.4% |
| sp13 | 60 | titin | RAGPVPPSFDQTIQDTQSNPGNLVRL  REVFLEDSGVFAVVAENRG | A0A5B7DEM6  MPC19546.1  *Portunus trituberculatus* | 71.78 | 118.9 | 14 | 3.4% |
| sp14 | 48 | / | / | / | / | / | 1 | 3.4% |
| sp15 | 43 | beta-actin | KSYELPDGQVITIGNERF | H9B3Y9  AFC88033.1  *Scylla paramamosain* | 41.8 | 30.25 | 1 | 3.4% |
| p16 | 42 | / | / | / | / | / | 1 | 3.4% |
| sp17-1 | 40 | arginine kinase | KLIDDHFLFKE  RIISMQMGGDLGQVYRR | H6VGI3  AFA45340.1  *Scylla paramamosain* | 40.3 | 104.8 | 3, 4, 10, 12, 13, 14, 17, 19, 20, 23, 27, 28, 29 | 44.8% |
| sp17-2 | 40 | tropomyosin | RIQLLEEDLERS | A0A6C0N3G5  QHW05413.1  *Scylla paramamosain* | 32.8 | 35.9 |  |  |
| sp18 | 36 | glyceraldehyde-3-phosphate dehydrogenase | KAGAEYVVESTGVFTTIEKA  KVVSWYDNEFGYSNRV | J7H926  AFP89956.1  *Scylla paramamosain* | 35.7 | 168.4 | 1 | 3.4% |
| sp19 | 28 | glutathione-s-transferase-like | RLNPQHTVPTLTEGDFALWESRA | A0A0P4WPH2  JAI63355.1  *Scylla olivacea* | 27.1 | 123.46 | 1 | 1 |
| sp20 | 22 | / | / | / | / | / | 1 | 3.4% |
| sp21-1 | 20 | myosin light chain | KFAFSIYDFEGKG  KVDDFLPIFAQVKK | A0A514C9K9  QDH76468.1  *Scylla paramamosain* | 17.4 | 153.5 | 4 | 3.4% |
| sp21-2 | 20 | muscle-specific protein 20 | KYGVPHEEIFQTADLFERR | A0A2P2HVY1  LAB65949.1  *Hirondellea gigas* | 19.7 | 136.3 |  |  |

#### **Table S2-4** Chionoecetes opilio

| Band | MW on SDS-PAGE (kDa) | Protein name | Identified peptide | Protein Accession number and Organism # | Protein MW  (kDa) | Score | Reactive serum | Sensitization rate |
| --- | --- | --- | --- | --- | --- | --- | --- | --- |
| co1 | >250 | / | / | / | / | / | 4 | 3.4% |
| co2 | >250 | / | / | / | / | / | 4, 5 | 6.9% |
| co3 | 223 | myosin heavy chain | RYPIYTNRT  KTLNSTQPHFIRC  RDLEETNIQQEAALGLLRK | A0A8J4XWP0  KAG0710435.1  *Chionoecetes opilio* | 219.6 | 125.47 | 4 | 3.4% |
| co4 | 200 | / | / | / | / | / | 1 | 3.4% |
| co5 | 150 | / | / | / | / | / | 4, 7 | 6.9% |
| co6-1 | 115 | paramyosin | RQAISLQEIQAHYDEIQRQ  RQLQTTLDQYGVAQRR | A0A8J4YAP8  KAG0719394.1  *Chionoecetes opilio* | 95.4 | 124.1 | 4 | 3.4% |
| Co6-2 | 115 | alpha-actinin | KGISQEQLNEFRS  RILAGDKPYILPEELQRE | A0A8J4YHK7  KAG0726703.1  *Chionoecetes opilio* | 95.5  103.2 | 94.73 | 4 | 3.4% |
| co7/8-1 | 115 | alpha-actinin | KGISQEQLNEFRS  RILAGDKPYILPEELQRE | A0A8J4YHK7  KAG0726703.1  *Chionoecetes opilio* | 95.5  103.2 | 94.73 | 4 | 3.4% |
| co7/8-2 | 100 | filamin C | RGEQGMPNEFNVWTRE  KFNGIHIPGSPFRL  KHVGHNNYQVGYVIRD | A0A8J4YG22  KAG0726642.1 *Chionoecetes opilio* | 92.1 | 146.1 | 4 | 3.4% |
| co9 | 95 | glycogen phosphorylase | RDYYFALANTVRD  KFFNDGDYIQAVLDRN | A0A0P4VYA7  JAI58945.1  *Scylla olivacea* | 97.8 | 488.25 | 1, 5 | 6.9% |
| co10 | 76 | hemocyanin | KLLMQELNDHRL  KYGGYFPSRPDKV  KDFTAEAVITNNNDHEVEATIRV  KYDNNHVEFSFNDGRW  RWNAIELDRFWTKL | A0A8J4YLL0  KAG0724994.1  *Chionoecetes opilio* | 74.8 | 258.1 | 3 ,4, 5, 12, 13, 19, 27 | 24.1% |
| co11 | 76 | hemocyanin | KLLMQELNDHRL  KYGGYFPSRPDKV  KDFTAEAVITNNNDHEVEATIRV  KYDNNHVEFSFNDGRW  RWNAIELDRFWTKL | A0A8J4YLL0  KAG0724994.1  *Chionoecetes opilio* | 74.8 | 258.1 | 4 | 3.4% |
| co12 | 48 | / | / | / | / | / | 27 | 3.4% |
| co13-1 | 40 | tropomyosin | KSQLVENELDHAQEQLSAATHKL  KAFANAEGEVAALNRR  RIQLLEEDLERS  KIVELEEELRV  KEVDRLEDELVNEKE | A2V735  BAF47267.1  *Chionoecetes opilio* | 32.7 | 328.2 | 3, 4, 9,10, 11, 12, 13, 14, 16, 17, 19, 20, 21, 22, 23, 25, 27, 28, 29 | 65.5% |
| co13-2 | 40 | arginine kinase | RIISMQMGGDLGQVFRR | A0A1P8D9V4  APU53308.1  *Penaeus japonicus* | 40.1  32 | 132.98 |  |  |
| co14 | 29 | / | / | / | / | / | 17, 27 | 6.9% |
| co15 | 28 | / | / | / | / | / | 27 | 3.4% |
| co16-1 | 20 | muscle-specific protein 20 | KYGVPHEEIFQTADLFERR | A0A8J4Y207  KAG0718948.1  *Chionoecetes opilio* | 17.9 | 97.25 | 4 | 3.4% |
| co16-2 | 20 | calponin | KQVSQVTTTLFALGRA | A0A8J4Y7E2  KAG0718949.1  *Chionoecetes opilio* | 19.1 | 42.5 |  |  |
| co16-3 | 20 | troponin C | KGYITPETVGTILRM | A0A8J5CR42  KAG0717811.1  *Chionoecetes opilio* | 11.8 | 63.7 |  |  |
| co16-4 | 20 | myosin light chain | KTDDFLPIFAQVKK | A0A8J5CLS5  KAG0725178.1  *Chionoecetes opilio* | 17.5 | 58.4 |  |  |

#### **Table S-5** Eriocheir sinensis

| Band | MW on SDS-PAGE (kDa) | Protein name | Identified peptide | Protein Accession number and Organism # | Protein MW  (kDa) | Score | Reactive serum | Sensitization rate |
| --- | --- | --- | --- | --- | --- | --- | --- | --- |
| es1 | >250 | / | / | / | / | / | 5 | 3.4% |
| es2 | 230 | / | / | / | / | / | 4 | 3.4% |
| es3 | 180 | glycogen debranching enzyme | RGYDELVPHHIHVVDEQRV  KDSHNASQFWADFQLRC | A0A0P4W3T4  JAI57852.1  *Scylla olivacea* | 226.9 | 150.9 | 1 | 3.4% |
| es4 | 150 | muscle M-line assembly protein | RVIADPKPTITWYRD  RVTATVTLEKEELYRI  KYVVQMEMIRA | A0A5B7CW41  MPC14057.1  *Portunus trituberculatus* | 89.48 | 111.2 | 4 | 3.4% |
| es5-1  es6 | 115 | alpha-actinin | KGISQEQLNEFRS  RTINEVENQILTRD  RILAGDKPYILPEELQRE | A0A8J4YHK7  KAG0726703.1  Chionoecetes opilio | 103.2 | 112.9 | 4 | 3.4% |
| es5-2 | 110 | calcium-transporting ATPase | KEFTLEFSRD | A0A6A4VBB0  KAF0287632.1  Amphibalanus *amphitrite* | 109.6 | 43.6 | 4 | 3.4% |
| es7 | 76 | hemocyanin | RKGENFFWVHHQLTVRF  KGENFFWVHHQLTVRF  KFDLPPGVLEHFETATRD  KYDNNGVEYSFNDGRW  KYHSGLGLPNRF  KYPDNRPHGYPLDRR | K4EJG5  AEG64817.1  *Eriocheir sinensis* | 78.3 | 125.79 | 5, 25, 27 | 10.3% |
| es8 | 63 | pyruvate kinase | KGVNLPGVPVDLPAVSEKDRS  KMGVDMVFASFIRD | A0A8J4Y0Q8  KAG0718033.1  *Chionoecetes opilio* | 82.5 | 91.82 | 5 | 3.4% |
| es9-1 | 40 | arginine kinase | RIISMQMGGDLGQVYRR  RVPFSHHDRL  RGEHTEAEGGIYDISNKR | Q9NH48  AAF43437.1  *Eriocheir sinensis* | 40.3 | 380.8 | 3, 4, 9, 10, 11, 12, 13, 14, 16, 17, 19, 20, 21, 22, 23, 27 | 55.2% |
| es9-2 | 40 | tropomyosin | RIQLLEEDLERS | A0A6C0N3G5  QHW05413.1  *Scylla paramamosain* | 32.8 | 35.9 |  |  |
| es10 | 29 | / | / | / | / | / | 1, 4, 17 | 10.3% |
| es11 | 25 | / | / | / | / | / | 10, 11, 16 | 10.3% |
| es12 | 22 | / | / | / | / | / | 17 | 3.4% |
| es13-1 | 20 | calponin | KQVSQVSTTLFALGRA | A0A0P4VWA4  JAI57033.1  *Scylla olivacea* | 20.2 | 87.2 | 4 | 3.4% |
| es13-2 | 20 | muscle-specific protein 20 | KYGVPHEEIFQTADLFERR | A0A8J4Y207  KAG0718948.1  *Chionoecetes opilio* | 22.1 | 75 |  |  |

#### **Table S2-6** Paralithodes camtschaticus

| Band | MW on SDS-PAGE (kDa) | Protein name | Identified peptide | Protein Accession number and Organism # | Protein MW  (kDa) | Score | Reactive serum | Sensitization rate |
| --- | --- | --- | --- | --- | --- | --- | --- | --- |
| pc1 | >250 | / | / | / | / | / | 4 | 3.4% |
| pc2 | >250 | / | / | / | / | / | 5 | 3.4% |
| pc3 | 223 | myosin heavy chain | RIEELEQEVEHERQ  RDLEETNIQQEAALGLLRK | A0A8J5N6U0  KAG7174238.1  *Homarus americanus* | 218.3  162.8 | 125.47 | 4, 5, 7 | 10.3% |
| pc4 | 200 | glycogen debranching enzyme | RDTFIALPGNLLITGRF | A0A8B7NGA3  /  *Hyalella azteca* | 180.8  226.9 | 31.36 | 6 | 3.4% |
| pc5 | / | / | / | / | / | / | 4, 5, 17 | 10.3% |
| pc6 | 100 | alpha-actinin | RVGWEQLLTSINRT  RTINEVENQILTRD  RENVDSDTAEQVIDSFRI  RILAGDKPYILPEELQRE | A0A8J4YHK7  KAG0726703.1  *Chionoecetes opilio* | 95.5  103 | 185.2 | 5 | 3.4% |
| pc7/8 | 76 | hemocyanin | RKGELFFWAHHQLTVRF  KFNLPPGVLEHFETATRD | A0A0U1ZZP8  AKC96432.1  *Scylla paramamosain* | 74.8  76.5 | 82.16 | 4, 5, 10, 12, 13, 14, 27 | 24.1% |
| pc9-1 | 40 | arginine kinase | RIISMQMGGDLGQVFRR | A0A1P8D9V4  APU53308.1  *Penaeus japonicus* | 40.1  32 | 132.98 | 3, 4, 7, 9, 10, 11, 12, 13, 14, 16, 17, 19, 20, 21, 22, 23, 25, 27, 28, 29 | 69.0% |
| pc9-2 | 40 | tropomyosin | KALQNAEGEVAALNRR  RIQLLEEDLERS  KIVELEEELRV | A0A6C0N3G5  QHW05413.1  *Scylla paramamosain* | 32.8  30.61 | 91 |  |  |
| pc10 | 35 | malate dehydrogenase | KDIDAAFLVGAMPRK  KNVIIWGNHSSTQFPDVRH  RIFGVTTLDIVRA  KAGAGSATLSMAYAGARF | A0A8J5CIV7  KAG0711979.1  *Chionoecetes opilio* | 35.5  35.5 | 86.24 | 3, 4, 10, 12, 13, 14, 16, 19, 20, 23, 25, 27 | 41.4% |
| pc11 | 28 | / | / | / | / | / | 4, 17 | 6.9% |
| pc12 | 20 | muscle-specific protein 20 | REWDEDQQRQ | A0A8J5JJ55  KAG7158191.1  *Homarus americanus* | 19.4  19.5 | 73.2 | 4 | 3.4% |
