# Supplementary material for: Revealing the Diverse Allergenic Protein Repertoire of Six Widely Consumed Crab Species: A Species‐Specific Allergen in King Crab
Source: Allergy. 2025 Jul 30;81(5):1500–21. doi: 10.1111/all.16674 (PMC13139819; doi:10.1111/all.16674)
Supplement: Supplementary file 4 — Table S3. Previously identified IgE‐binding linear epitopes of four crab allergens. [file ALL-81-1500-s002.docx]

**Table S3.** Previously identified IgE-binding linear epitopes of four crab allergens. # Allergens (PM, paramyosin; MHC, myosin heavy chain; TM, tropomyosin; FLNC; filamin C; AK, arginine kinase; GP, glycogen phosphorylase; ALD, aldolase; MDH, malate dehydrogenase; PGM, phosphoglucomutase; HSP, heat shock protein; HC, hemocyanin) * Crab species of the allergen protein (CF, *Charybdis feriata*; PP: Portunus pelagicus; SP, *Scylla paramamosain*; CO, *Chionoecetes opilio*; ES, *Eriocheir sinensis*; PC, *Paralithodes camtschaticus*)

| Allergen# | Species* | No. | Start-end | Amino acid sequence |
| --- | --- | --- | --- | --- |
| TM | SP | TM-E1 | 2-9 | DAIKKKMQ |
|  |  | TM-E2 | 19-29 | MDRADTLEQQN |
|  |  | TM-E3 | 39-55 | TEEEIRATQKKMQQVEN |
|  |  | TM-E4 | 59-70 | AQEQLSAANTKL |
|  |  | TM-E5 | 76-90 | ALQNAEGEVAALNR |
|  |  | TM-E6 | 99-233 | LERSEERLNTATTKLAEASQAADESERMRKVLENRSLSDEERMDALENQLKEARFLAEEADRKYDEVARKLAMVEADLERAEERAESGESKIVELEEELRVVGNNLKSLEVSEEKANQREETYKEQIKTLANKLK |
|  |  | TM-E7 | 253-264 | VDRLEDELVNEK |
|  |  | TM-E8 | 275-283 | LDQTFSELSG |
| FLNC | SP | FLNC-E1 | 28–43 | EGLHELHVKYNAEHVQ |
|  |  | FLNC-E2 | 151–165 | GEGRKRNQISVGSQS |
|  |  | FLNC-E3 | 187–205 | APSGLEEPCFLKKLPNGHL |
|  |  | FLNC-E4 | 257–273 | EGQTHKENQFTIDTRDA |
|  |  | FLNC-E5 | 321–353 | NHVPGSPFTVKVTGEGTNRQTERIKRQREAVPL |
|  |  | FLNC-E6 | 363–377 | TFKLPGISPFDLGAT |
|  |  | FLNC-E7 | 418–433 | EMHIPGSPFQFTVGPL |
|  |  | FLNC-E8 | 446–462 | PGLERGEQGMPNEFNVW |
|  |  | FLNC-E9 | 490–505 | DGSCYVSYVVAEPGEY |
|  |  | FLNC-E10 | 512–528 | NDKHIPDSPYKVYITPS |
|  |  | FLNC-E11 | 615–632 | PFRLRIGKDEADPAAVSV |
|  |  | FLNC-E12 | 646–660 | TDFIVDTCNAGAGTL |
|  |  | FLNC-E13 | 726–741 | ESSSVVVETVEKTKSG |
|  |  | FLNC-E14 | 743–761 | KGHHGTIIPKFHSDANKVT |
|  |  | FLNC-E15 | 817–828 | SYKVKERGNHIL |
| AK | SP | AK-E1 | 113-155 | VDPDGKFVISTRVRCGRSMEGYPFNPCLTEAQYKEMESKVSST |
|  |  | AK-E2 | 204–225 | WPTGRGIYHNDNKTFLVWCNEE |
|  |  | AK-E3 | 316-330 | EAEGGVYDISNKRR |
| HC | ES | HC-E1 | 167-202 | NSEVIQEAYTAQMTQTPSKIKSHFTGSKSNPEQR |
|  |  | HC-E2 | 237-255 | FWWDDSHENHHIERKGENF |
|  |  | HC-E3 | 331-350 | IRDAIAHGYITAKDGSTISI |
|  |  | HC-E4 | 360-378 | GDVIESSTYSPNPQYYGAL |
|  |  | HC-E5 | 381-401 | TAHVMLGRQGDPHGKFDLPP |
|  |  | HC-E6 | 423-442 | DNIFREHKDSLTPYTTQELE |
|  |  | HC-E7 | 501-510 | VTNNNGKEVS |
|  |  | HC-E8 | 512-534 | TVRAFAWPKYDNNGVEYSFNDGR |
|  |  | HC-E9 | 547–572 | LSPGANTITRSGKDSAVTVPDVPSFK |
|  |  | HC-E10 | 593–502 | KYHSGLGLPNR |
|  |  | HC-E11 | 634-662 | SDGEEDATVDGLHDSTSFNHYGCADGKYPDNRPHGYPLDRR |
